# Re‐evaluating the actin‐dependence of spectraplakin functions during axon growth and maintenance

**DOI:** 10.1002/dneu.22873

**Published:** 2022-04-22

**Authors:** Yue Qu, Juliana Alves‐Silva, Kriti Gupta, Ines Hahn, Jill Parkin, Natalia Sánchez‐Soriano, Andreas Prokop

**Affiliations:** ^1^ Manchester Academic Health Science Centre Faculty of Biology Medicine and Health School of Biology, The University of Manchester Manchester UK; ^2^ Department of Molecular Physiology & Cell Signalling Institute of Systems Molecular & Integrative Biology University of Liverpool Liverpool UK; ^3^ Present address: Division of Nutritional Sciences, College of Human Ecology, Cornell University, Ithaca, USA; ^4^ Present address: Departamento de Morfologia, Instituto de Ciências Biológicas, Universidade Federal de Minas Gerais, Belo Horizonte, Brazil

**Keywords:** actin, axons, *Drosophila*, microtubules, neurons

## Abstract

Axons are the long and slender processes of neurons constituting the biological cables that wire the nervous system. The growth and maintenance of axons require loose microtubule bundles that extend through their entire length. Understanding microtubule regulation is therefore an essential aspect of axon biology. Key regulators of neuronal microtubules are the spectraplakins, a well‐conserved family of cytoskeletal cross‐linkers that underlie neuropathies in mouse and humans. Spectraplakin deficiency in mouse or *Drosophila* causes severe decay of microtubule bundles and reduced axon growth. The underlying mechanisms are best understood for *Drosophila*’s spectraplakin Short stop (Shot) and believed to involve cytoskeletal cross‐linkage: Shot's binding to microtubules and Eb1 via its C‐terminus has been thoroughly investigated, whereas its F‐actin interaction via N‐terminal calponin homology (CH) domains is little understood. Here, we have gained new understanding by showing that the F‐actin interaction must be finely balanced: altering the properties of F‐actin networks or deleting/exchanging Shot's CH domains induces changes in Shot function—with a Lifeact‐containing Shot variant causing remarkable remodeling of neuronal microtubules. In addition to actin‐microtubule (MT) cross‐linkage, we find strong indications that Shot executes redundant MT bundle‐promoting roles that are F‐actin‐independent. We argue that these likely involve the neuronal Shot‐PH isoform, which is characterized by a large, unexplored central plakin repeat region (PRR) similarly existing also in mammalian spectraplakins.

## 1 INTRODUCTION

Axons are the slender, up to 2 m long processes of neurons that form the biological cables wiring our bodies (Prokop, 2020). Their de novo formation during development, regeneration, or brain plasticity is implemented at growth cones (GCs), the amoeboid tips of extending axons (Harrison, 1910; Ramón y Cajal, 1890). GCs navigate by sensing spatiotemporally patterned chemical and mechanical cues along their paths which are translated into orchestrated morphogenetic changes leading to axon extension (Franze et al., 2013; Sanes et al., 2019; Tessier‐Lavigne & Goodman, 1996).

These morphogenetic changes are mediated by the cytoskeleton, in particular, actin and microtubules (MTs; Dent et al., 2011; Lowery & van Vactor, 2009; Prokop et al., 2013; Tanaka & Sabry, 1995): F‐actin in the GC periphery is required for explorative protrusive activity and mechano‐transduction leading to the directional stabilization of MTs which, in turn, implement the actual growth events (e.g., Buck & Zheng, 2002; Geraldo et al., 2008; A. C. Lee & Suter, 2008; Qu et al., 2019; Suter & Forscher, 2001). When MTs in GCs arrange into bundled loops or spools, they seem to suppress such interactions in the periphery and slow down axon growth (Dent et al., 1999).

The MTs of GCs originate from the MT bundles of the axon shaft. These bundles are fairly loose but run all along axons and serve as the essential highways for axonal transport (Prokop, 2020). They must therefore be maintained throughout an organism's lifetime, which requires active maintenance, repair, and turnover (Hahn et al., 2019; Prokop, 2021). These bundles can also drive axon elongation through so‐called intercalative or stretch growth (Bray, 1984; Lamoureux et al., 2010; Smith, 2009; Zheng et al., 1991). For this, axons display forward drift of MT bundles (Miller & Sheetz, 2006; Roossien et al., 2013) or MT sliding forces (Lu et al., 2015; Winding et al., 2016). Like in GCs, the MT bundle regulation in axon shafts requires actin–MT interactions, which is required for their parallel arrangements and to uphold MT numbers (Alves‐Silva et al., 2012; Datar et al., 2019; Krieg et al., 2017; Qu et al., 2017).

**FIGURE 1:**
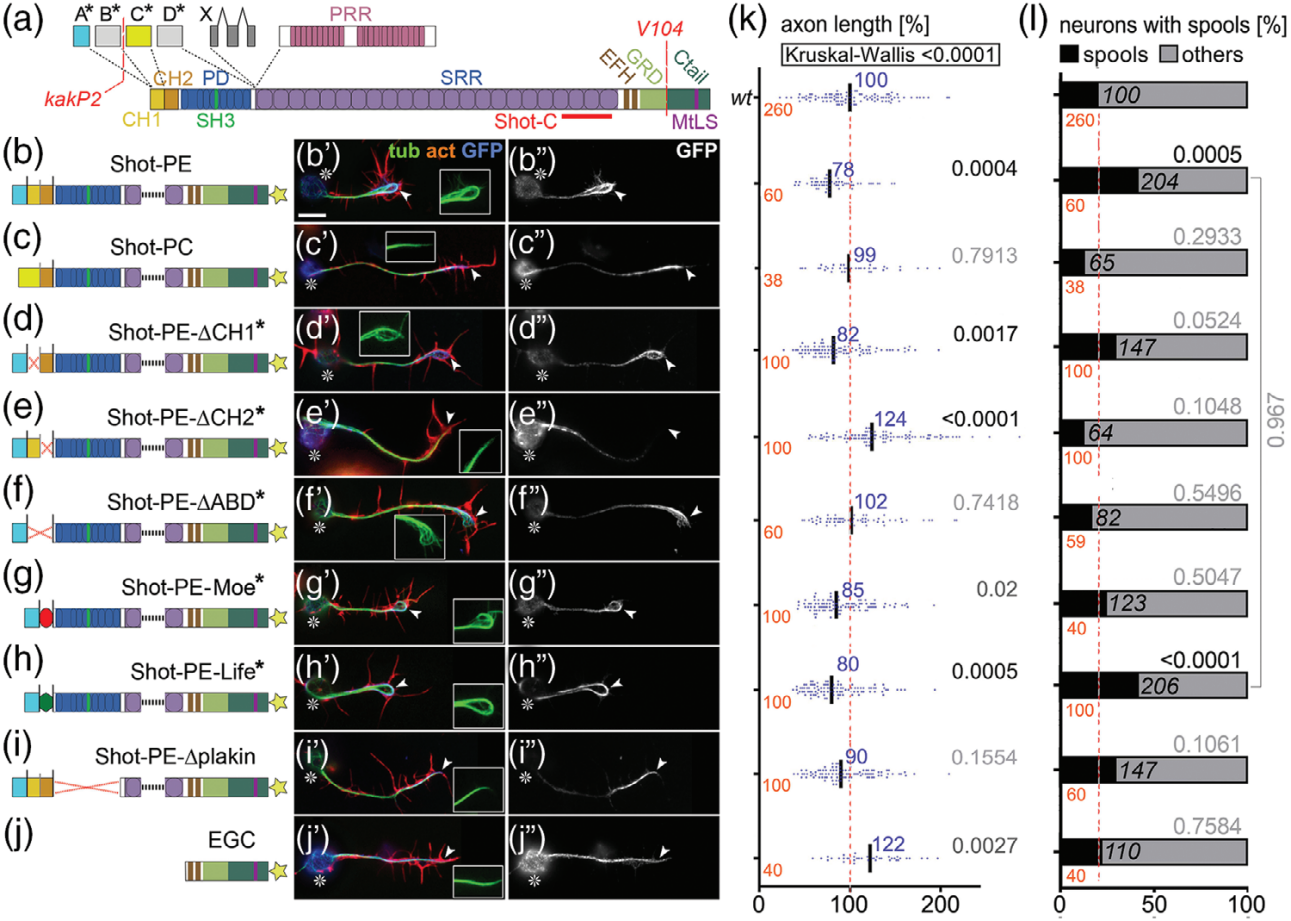
Different Shot constructs and their localization. (a) Illustration of different Shot isoforms as a function of different start sites (A*–D*) and splice‐in of different exons (X, PRR); different domains and motifs are color‐coded (CH, calponin homology; PD, plakin domain; PRR, plakin repeat region; SRR, spectrin repeat region; EFH, EF‐hand; GRD, Gas2‐related domain; MtLS, MT tip localization sequence which forms the Eb1‐binding motifs); positions of the epitope used to generate the Shot‐C antibody (Strumpf & Volk, 1998), the *kakP2* P‐element insertion (blocking the a* and b* start sites) and the break‐point of the *V104* inversion (deleting the Ctail) are indicated in red. (b–j) Different UAS‐constructs expressing modified Shot versions, color‐coded as in (A) and GFP indicated by a yellow star; newly generated constructs are indicated by asterisks, origins of all other constructs are provided in the Methods section. (b’–j’) Primary neurons at 6–8 h in vitro (HIV) cultured on glass which express the respective constructs on the left and are stained for actin (red), tubulin (green), and GFP (blue); wild‐type reference neurons are not shown but can take on any of the shapes displayed in B’‐J’ (see examples in Figures 2a, 4b, 6a, 8a, 8g, and Figure S1a). (b″–j″) GFP channel are shown in grayscale. In all images, asterisks indicate cell bodies, arrow heads the axon tips; scale bar in (A) represents 10 μm in all images. (k and l) Graphs display the distribution of axon length phenotypes (K) and frequency of spools in neuronal growth cones (GCs) (L) taken from neuron populations expressing the same constructs as displayed in b″–j″. Number of neurons analyzed are shown in orange, median values in blue (k only), black numbers within columns in (L) indicate the percentage of neurons with spool‐containing GCs; black/gray numbers on the right of each plot/bar indicate the *p*‐values obtained via Mann–Whitney rank sum tests in (K) (Kruskall–Wallis analysis of variance [ANOVA] test results shown above) and chi‐square tests in (l). Data were normalized to wild‐type controls performed in parallel to all experiments (red dashed lines)

Numerous mechanisms have been described that mediate actin–MT interaction (Dogterom & Koenderink, 2019; Kundu et al., 2021; Mohan & John, 2015). In axons, very prominent mediators are the spectraplakins, an evolutionarily well‐conserved family of multi‐domain cytoskeletal linker proteins (Figure 1a; Voelzmann et al., 2017). Of these, dystonin was discovered in a mouse model of sensory neuropathy, later shown to involve severe MT bundle deterioration and be linked to human hereditary sensory and autonomic neuropathy (HSAN6: OMIM #614653; Dalpe et al., 1998; Duchen et al., 1964; Edvardson et al., 2012; Eyer et al., 1998). Its mammalian paralogue ACF7/MACF1 was discovered as an actin–MT cross‐linker (Byers et al., 1995; Leung et al., 1999), later shown to be involved in neuronal development (Goryunov et al., 2010; Ka et al., 2014; Ka & Kim, 2015; Sánchez‐Soriano et al., 2009) and linked to lissencephaly (OMIM #618325). As detailed elsewhere (Voelzmann et al., 2017), spectraplakins act as actin–MT cross‐linkers by binding F‐actin via a tandem of N‐terminal calponin homology domains (CH domains) and associate with MTs through their C‐terminus; this C‐terminus harbors a Gas2‐related domain (GRD) which also stabilizes MTs against depolymerization, and a positively charged unstructured Ctail which also binds to Eb1 (Figure 1a; Alves‐Silva et al., 2012; Goriounov et al., 2003; Honnappa et al., 2009; S. Lee & Kolodziej, 2002).

The *Drosophila* spectraplakin Short stop (Shot) is a close orthologue of dystonin and ACF7/MACF1; in neurons, Shot is required for axon and dendrite growth, neuronal polarity, axonal compartmentalization, synapse formation, and axonal MT bundle maintenance (S. Lee et al., 2000; Prokop et al., 1998; Reuter et al., 2003; Voelzmann et al., 2017). In Shot‐deficient neurons, MT bundles in axon shafts and GCs frequently disintegrate into disorganized, curled, criss‐crossing arrangements (from now on referred to as MT curling). This dramatic MT phenotype can be rescued when reinstating actin–MT cross‐linking activity of Shot, through a mechanism where Shot guides the extension of polymerizing MTs along the axonal cortex into parallel bundles (Alves‐Silva et al., 2012; Hahn et al., 2021; Sánchez‐Soriano et al., 2010). The C‐terminal MT interaction involved in this function of Shot is quite well described. In contrast, little is known about Shot's N‐terminal interaction with neuronal F‐actin networks; for example, how it is influenced by different forms of F‐actin networks which can present as sparse cortical F‐actin rings in the axon shaft (Leterrier et al., 2017; Qu et al., 2017; Xu et al., 2013) or dense lattice‐like or bundle‐forming F‐actin networks in GCs (Dent et al., 2011).

Here, we have gained new understanding of Shot's F‐actin interaction. First, we show that Shot function does not simply depend on F‐actin: it rather appears to involve a well‐balanced interplay of low‐affinity CH domains with F‐actin networks, where any changes can trigger alterations in Shot's functional output; this phenomenon directs the formation of MT spools relevant for axon growth regulation. In the axon shaft, Shot is required for MT bundle maintenance through the above‐mentioned guidance mechanism depending on F‐actin/MT/Eb1 cross‐linkage. In addition, we provide strong indications that Shot performs actin‐independent bundle‐maintaining functions that act redundantly to F‐actin/MT/Eb1 guidance. We argue that these functions are mediated by the neuronally enriched Shot‐PH isoform. Shot‐PH is the only isoform displaying an evolutionarily conserved plakin repeat region (PRR; Hahn et al., 2016; Röper & Brown, 2003; Voelzmann et al., 2017), which is functionally unexplored and might hold the key to uncharted mechanisms of axon biology and architecture.

## 2 RESULTS

### 2.1 Roles of Shot's actin‐binding domain in gain‐of‐function experiments

**FIGURE 2:**
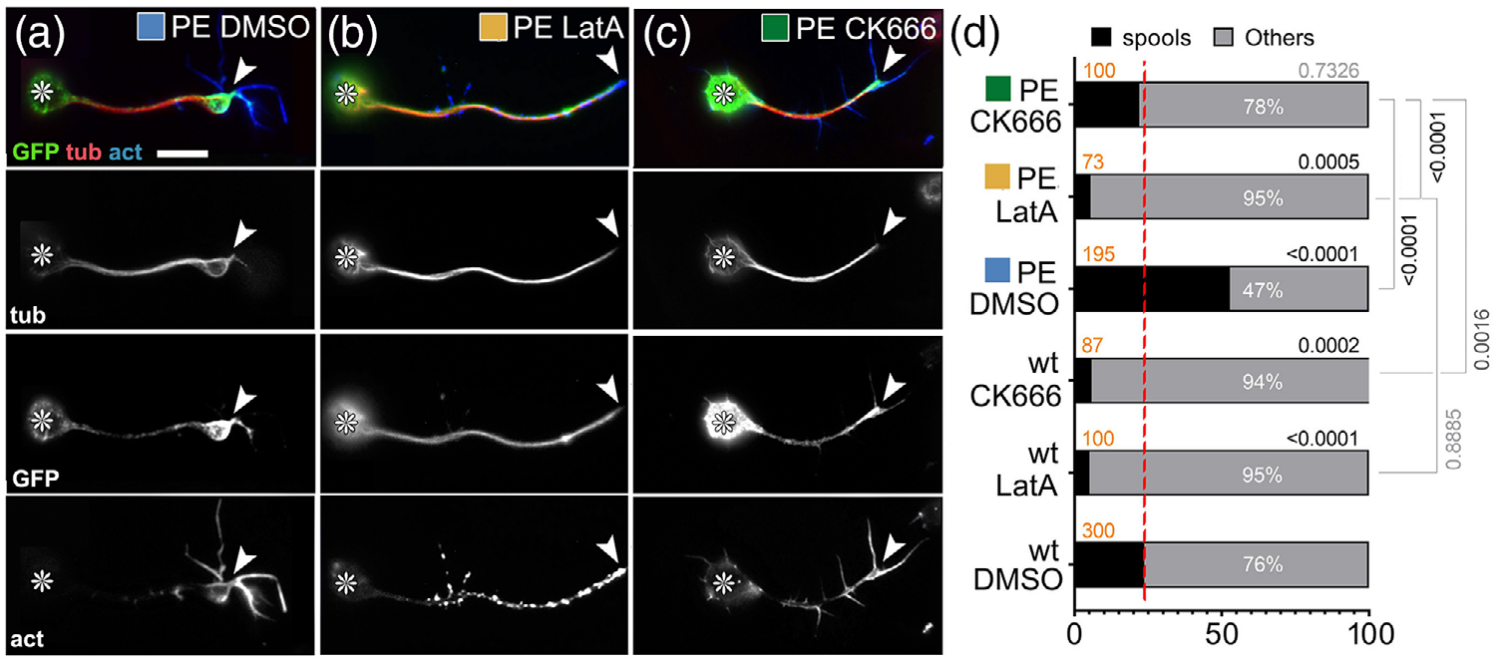
Impact of drug‐induced F‐actin inhibition on Shot‐PE function. (a–c) Primary neurons at 6–8 h in vitro (HIV) on glass treated with DMSO (control), LatA or CK666 as indicated and stained for GFP (green), tubulin (red), and actin (blue); grayscale images below show single channels as indicated; asterisks indicate cell bodies, arrowheads the tips of axons; scale bar in (a) represents 10 μm in all images. (d) Frequency of neurons with growth cones (GCs) that contain spools (examples of neurons in a–c are assigned to their respective data columns via color‐coded squares); orange numbers indicate the sample numbers (number of neurons analyzed), white numbers within columns the percentage of neurons with GCs that contain no spools; numbers on the right of each graph indicate the *p*‐values obtained via chi‐squared tests. Data were normalized to wild‐type controls performed in parallel to all experiments (dashed red line)

To assess F‐actin dependency of Shot function, we first took a gain‐of‐function (GOF) approach. For this, we targeted the expression of transgenic Shot constructs to primary *Drosophila* neurons which were fixed after 6 h in vitro (HIV) and analyzed for two phenotypes: we quantified the length of axons and the number of neurons showing growth cones with bundled loops (referred to as ‘‘spools’’; Figure 1b’,g’,h’)—as opposed to GCs with ‘‘pointed’’ (Figure 1c’,i’,j’) or ‘‘disorganized’’ MTs (Figure 1f’; Sánchez‐Soriano et al., 2010; Teng et al., 2001). Neuronal expression of Shot‐PE::GFP (a GFP‐tagged version of the best‐studied PE isoform; Hahn et al., 2016; Figure 1a,b) caused a reduction in axon length at fixation stage to ∼80% and doubled the number of MT spools in GCs compared to wild‐type controls (Figure 1b’,k,l). In contrast, Shot‐PC::GFP (another natural isoform which lacks CH1; Figure 1a,c) failed to induce either of these phenotypes; instead it showed a slight trend to suppress spool numbers below control levels (Figure 1c,l), as observed in previous studies (Sánchez‐Soriano et al., 2010). The finding suggests that an interaction with F‐actin is essential for spool formation, since the lack of CH1 in the Shot‐PC isoform (Figure 1c) eliminates F‐actin interaction (concluded from previous localization and binding studies; S. Lee & Kolodziej, 2002). Accordingly, spool induction can also be suppressed when depleting F‐actin with the drug latrunculin A (LatA; Figure 2b,d; Sánchez‐Soriano et al., 2010).

Shot‐PE and Shot‐PC not only differ in the presence/absence of CH1 they also display different lead sequences that flank CH domains N‐terminally (blue A* vs. yellow C* in Figure 1a–c; Hahn et al., 2016). Both lead sequences lack any informative homologies or motifs but may still have modifying impacts on CH domain functions (Yin et al., 2020). Therefore, we generated Shot‐PE variants containing the A* lead sequence but lacking single or both CH domains (Figure 1d–f). When expressing these variants, Shot‐PE‐ΔABD::GFP (lacking both CH domains) caused phenotypes almost identical to those of Shot‐PC (Figure 1f’,k,l), thus corroborating former claims that the actin‐binding capability of Shot‐PC is negligible (S. Lee & Kolodziej, 2002).

In contrast, single CH domain deletions generated surprising results. As mentioned above, CH1 is the main actin‐binding domain of the tandem, and we expected therefore that Shot‐PE‐ΔCH2 would have actin‐binding, hence spool‐inducing, capability whereas Shot‐PE‐ΔCH1 would behave like Shot‐PC or Shot‐ΔABD. However, the opposite was true: deleting the functionally less prominent CH2 caused robust axon elongation to ∼120% and failure to induce extra spools, suggesting that the CH1 domain alone fails to mediate actin‐binding properties (Figure 1e,k,l). In contrast, Shot‐PE‐ΔCH1 expression had a trend toward extra spool formation and shorter axons, suggesting modest actin‐binding properties although CH1 was absent (Figure 1d’,k,l). Since Shot‐PE‐ΔCH1 and Shot‐PC only differ in the presence of either the A* or the C* lead sequence (Figure 1c,d), our results might hint at potential regulatory roles: for example, the C* lead sequence of Shot‐PC might inhibit residual actin affinities of CH2 but not the A* sequence of Shot‐PE, thus explaining why Shot‐RE‐ΔCH1 seemed to display more activity than Shot‐RC and Shot‐RE‐ΔABD.

### 2.2 F‐actin is required for Shot construct localization

To gain more understanding of these phenotypes, we performed localization studies. Shot‐PE::GFP was strongly enriched at the distal ends of axons, mostly at the actin‐enriched GCs; this was consistent with its spool‐inducing activity (Figure 1b″). Also, Shot‐PC::GFP and Shot‐PE‐ΔABD::GFP were distally enriched in axons (Figure 1c″,f″), suggesting that their inability to induce spools was not due to their physical absence but rather their functional impairment.

Also, Shot‐PE‐ΔCH1::GFP was enriched in distal axon segments (Figure 1d″). This localization was consistent with its spool‐inducing tendencies (potentially mediated by residual F‐actin affinity of CH2; see above). In contrast, the Shot‐PE‐ΔCH2::GFP construct was retained at or actively localized to proximal axon segments (Figure 1e’), consistent with the absence of its spool‐inducing activity (Figure 1l).

Taken together, except Shot‐PE‐ΔCH2::GFP all Shot constructs localized distally, including those lacking the F‐actin‐binding CH domains. This seemed to contradict further findings that Shot‐PE::GFP lost its tip localization upon removal of F‐actin with LatA (‘‘GFP’’ in Figure 2b)—similarly observed also with the F‐actin‐inhibiting drug cytochalasin D (CytoD; Figure S1b).

This actin dependence in the absence of CH domains did not involve C‐terminal domains of Shot: the GFP‐tagged C‐terminus (Shot‐EGC::GFP; comprising EF‐hand motifs and the MT‐binding GDR and Ctail; Figure 1j) localized homogeneously along axonal MTs, and did not induce extra spools or axon shortening (Figure 1j–l; Alves‐Silva et al., 2012). Instead, we focused on the N‐terminal plakin domain because Shot‐PE‐Δplakin::GFP had been reported to display transient localization defects in developing embryonic motor nerves (Bottenberg et al., 2009). However, like most other constructs, Shot‐PE‐Δplakin::GFP displayed distal localization in primary neurons (Figure 1i″), but it failed to induce robust spool formation or axon shortening (Figure 1k,l; consistent with its partial deficits in supporting axon growth in vivo; Bottenberg et al., 2009).

Taken together, our data suggest complex regulations at the N‐terminus. We propose that two domains can mediate F‐actin association: CH domains through direct binding, and the plakin domain (which contains a SRC Homology three motif of protein interaction; ‘‘SH3’’ in Figure 1a) through association with independent factors that are localized at GCs through F‐actin (e.g., transmembrane proteins; see Discussion). In this scenario, distal localization of Shot could be mediated by either the CH domains or the plakin domain alone, but its spool‐inducing function would depend on both domains in parallel; this would explain why single deletion of either the plakin or the CH domains abolishes Shot's spool‐inducing activity but not its localization.

### 2.3 Qualitative or quantitative changes of F‐actin interaction influence Shot's MT‐regulating roles

As explained above, we propose that Shot interacts with F‐actin networks through both the plakin and CH domains. This raises the question of whether Shot uses F‐actin as a mere anchor or whether its function is influenced by changes in the quantity and quality of F‐actin networks. To address this, we first introduced quantitative and qualitative changes to F‐actin networks by manipulating actin nucleation, that is, the process of seeding new actin filaments.

In *Drosophila* primary neurons, nucleation is performed primarily by the formin DAAM and the Arp2/3 complex (Gonçalves‐Pimentel et al., 2011; Prokop et al., 2011); of these, Arp2/3 is expected to contribute branched networks that are qualitatively different from those nucleated by formins (Blanchoin et al., 2014). Arp2/3‐mediated actin nucleation can be specifically inhibited by CK666 (Hetrick et al., 2013). When applying 100 nM CK666 for 2 h, we observed a reduction in filopodia numbers to 72 ± 5% (*p*_Mann–Whitney _< .001, *n* = 80), indicating successful Arp2/3 inhibition and a reduction in F‐actin abundance (Gonçalves‐Pimentel et al., 2011). Under these conditions, Shot‐PE::GFP was still recruited to the distal axon, but its spool‐inducing activity was strongly suppressed (Figure 2c,d). This finding supports our hypothesis that quantitative and/or qualitative changes of F‐actin networks impact MT regulatory roles of Shot.

To further challenge this notion, we decided to exchange the two CH domains of Shot for conceptually different actin‐binding domains taken from other proteins. For this, we chose the 17 residue actin‐binding motif Lifeact (Life) from the *Saccharomyces cerevisiae* protein Abp140 (Riedl et al., 2008), and the C‐ERMAD domain of Moesin (Moe; Kiehart et al., 2000; Millard & Martin, 2008). When extrapolating from binding studies reported for CH domains of α‐actinin (closely related to those of Shot; Figure S2), we expected that Shot's CH domains bind F‐actin modestly, whereas Life should bind F‐actin more robustly in a phalloidin‐like manner (Lemieux et al., 2014). In contrast, Ezrin's actin‐binding domain (closely related to Moe; Fritzsche et al., 2013, 2014) had been shown to dissociate even faster from F‐actin than α‐actinin's CH domains, consistent with observations that full‐length Moesin does not strongly co‐localize with F‐actin in embryonic chick neurons or PC12 cells (Amieva & Furthmayr, 1995; Marsick et al., 2012). We, therefore, predicted a gradual impact of the different actin‐binding domains on Shot localization and/or function in the hierarchical sequence Life > Shot CH1+2 ≥ Moe.

We first analyzed the localization of the different actin‐binding domains fused to the N‐terminal lead sequence of Shot‐PE (GFP::A*::CH1+2, GFP::A*::Life, GFP::A*::Moe; Figure S3) by transfecting them into *Drosophila* primary neurons. Like GFP controls, also GFP::A*::CH1+2 and GFP::A*::Moe were distributed fairly homogeneously throughout entire neurons, consistent with their expected low affinity for F‐actin (Figure S3a–c). In contrast, GFP::A*::Life showed the expected robust, phalloidin‐like staining (Figure S3d). None of the three fusion constructs caused any obvious MT phenotypes (Figure S3e).

**FIGURE 3:**
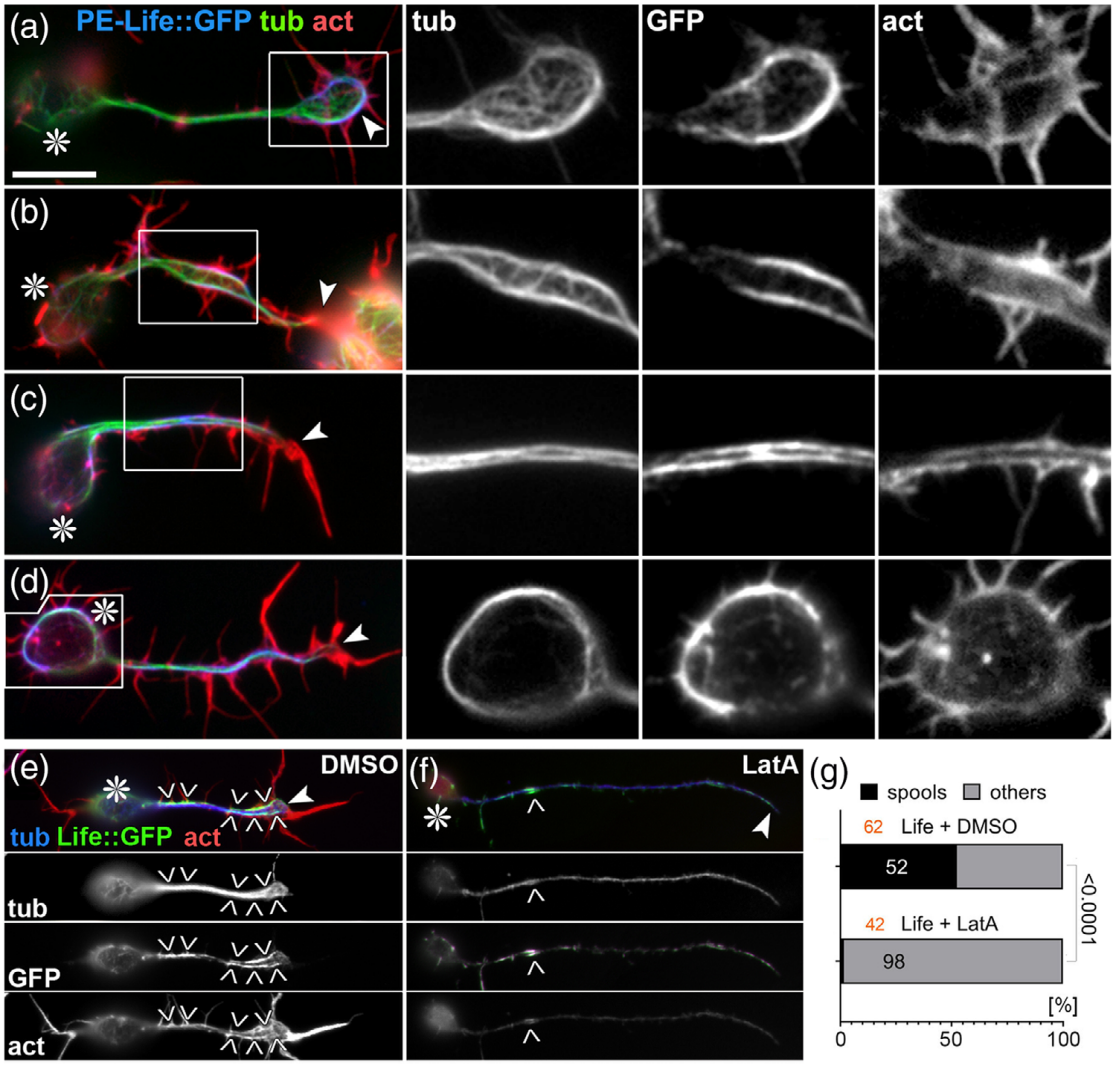
Characteristic phenotypes induced by Shot‐PE‐Life::GFP expression. (a–d) Primary neurons at 6–8 h in vitro (HIV) on glass with *scabrous‐Gal4*‐induced expression of Shot‐PE‐Life::GFP, stained for tubulin (green), actin (red), and GFP (blue); boxed areas are shown as twofold magnified single channel grayscale images on the right, as indicated. (e and f) Shot‐PE‐Life::GFP‐expressing neurons treated with vehicle (e) or latrunculin A (LatA; f), stained for the same markers as above but color‐coded differently (as indicated); grayscale images below show single channels. Asterisks in (a–f) indicate cell bodies, arrowheads tips of axons, chevrons in (e and f) indicate areas of high GFP concentration, and the scale bar in (a) represents 10 μm in all RGB images of (a–d), 5 μm in grayscale images of (a–d), and 20 μm in (e). (g) Percentage of Shot‐PE‐Life::GFP‐expressing neurons showing spools (black) when treated with vehicle or LatA; number of analyzed neurons in orange, percentage shown in bars, the chi‐squared test result on the right

We next replaced both CH domains in Shot‐PE::GFP with Life or Moe (Figure 1g,h) and generated transgenic flies using the same genomic landing site as utilized for other transgenic constructs in this study (see Methods); this makes sure that the expression strength was comparable between constructs (Bischof et al., 2007). When targeted to primary neurons, Shot‐PE‐Moe::GFP behaved like the ΔCH1 and Δplakin constructs: it was enriched along MTs in distal axons accompanied by mild axon shortening and a trend toward increased spool formation (Figure 1g″,k,l). In contrast, Shot‐PE‐Life::GFP localized strongly in GCs but also along axons (Figures 1h″ and 3; Figure S4) and caused axon shortening and spool induction to similar degrees as Shot‐PE::GFP (Figure 1k,l). However, other subcellular features were strikingly novel: (1) 38% of Shot‐PE‐Life::GFP‐induced MT spools in GCs had a ‘‘tennis racket’’ appearance with many MTs projecting diffusely through the center of spools (Figure 3a and ‘‘white arrows’’ in Figure S4); (2) a number of neurons showed unusual MT bundles in close proximity to the cortex in the cell bodies (Figure 3d and ‘‘open curved arrows’’ in Figure S4); (3) about 60% of axonal MT bundles were split into two parallel portions that were decorated with strong Shot‐PE‐Life::GFP staining, and closely accompanied by F‐actin staining that was unusually strong for axon shafts (Figure 3b,c and ‘‘white arrowheads’’ in Figure S4); these constellations suggested that the hybrid construct firmly cross‐links and alters the sub‐cellular arrangement of MTs and F‐actin while taking on an unusual localization itself (Figure 3 and Figure S4; see Discussion). The aberrant localization of Shot‐PE‐Life::GFP and its dominant MT phenotypes were clearly abolished when treating neurons with LatA, thus demonstrating the F‐actin dependence even of this powerful hybrid construct (Figure 3e–g).

Taken together, our GOF analyses suggested that the quality and quantity of F‐actin networks can regulate Shot's MT bundle‐inducing function. The low affinity of Shot's CH domains seems ideally tuned to read those differences in F‐actin: high abundance of F‐actin induces spools in GCs, while increases in Shot's F‐actin affinity (Shot‐PE‐Life) cause MT rearrangements (split bundles) even in axon shafts where F‐actin networks are usually sparse (Leterrier et al., 2017; Qu et al., 2017; Xu et al., 2013).

### 2.4 Shot's axon length regulation involves MT spool formation in GCs and MT bundle maintenance

**FIGURE 4:**
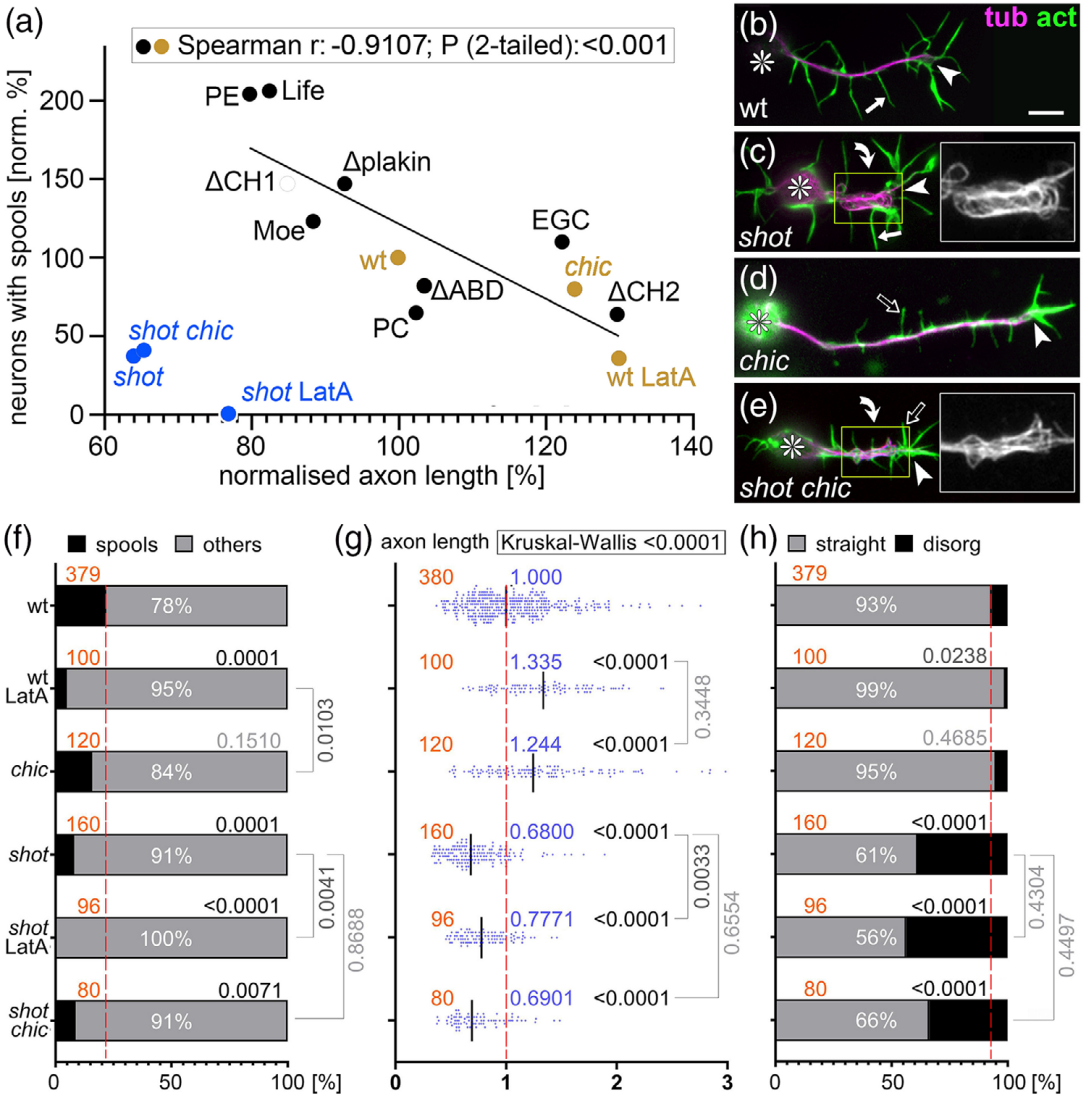
Microtubule (MT) loops correlate with axon lengths, but Shot has additional axon shaft phenotypes. (a) Spearman correlation analysis comparing axon length and spool frequency. Black dots represent data from Figure 1k plotted against data from Figure 1l, and orange/blue dots match data from (f) and (g); significant negative correlation (*r*‐ and *p*‐values) for orange and black dots are shown in box at top. (b–e) Primary neurons at 6–8 h in vitro (HIV) on glass which are either wild‐type (b), *shot^3/3^* (c), *chic^221/221^* (d) or *shot^3/3^ chic^221/221^* (e), stained for tubulin (magenta) and actin (green); asterisks indicate cell bodies, arrowheads tips of axons, curved arrows areas of MT curling, and white/open arrows normal/short filopodia (see quantifications in Figure S5); yellow‐boxed areas presented as twofold magnified insets showing the tubulin channel in grayscale; the scale bar in (b) represents 10 μm in all RGB images and 5 μm in insets. (f–h) Quantification of neurons displaying MT spools in growth cones (GCs; f), of axon lengths (g), and of neurons displaying MT curling in axonal shafts (h); numbers of analyzed neurons are indicated in orange; median values in blue (g), percentages as white numbers within columns (f and h); *p*‐values obtained via Mann–Whitney rank sum tests (g) or chi‐squared tests (f and h) are shown in black/gray above bars or plotted data; all data were normalized to wild‐type controls performed in parallel to all experiments (dashed red lines)

Our key readout for Shot GOF was the formation of MT spools in GCs. MT spools have been suggested to inhibit axon growth (Dent et al., 1999; Sánchez‐Soriano et al., 2010). Accordingly, we found a strong negative correlation between spools and axon lengths when plotting the data from our overexpression experiments (black dots in Figure 4a); also neurons without Shot GOF plotted onto this curve (Figure 4f,g and orange dots in [a]), including untreated wild‐type neurons, neurons treated with LatA (less spools, enhanced axon length), or neurons lacking the F‐actin‐promoting factor Chickadee (Chic, the sole profilin in *Drosophila*; Gonçalves‐Pimentel et al., 2011; slightly less spools, modest increase in axon length). Also, spool formation in neurons without Shot GOF seems to be mediated by Shot, as was suggested by *shot* mutant neurons where spool numbers were strongly reduced compared to wild‐type (Figure 4f; Sánchez‐Soriano et al., 2010).

However, *shot* mutant neurons do not plot onto the correlation curve (blue dots in Figure 4a): instead of showing axon extension that would usually correlate with the absence of spools, their axons were very short. Furthermore, combinatorial studies revealed that the short axon phenotype of *shot* overrides LatA‐ or *chic*‐induced axon elongation (Figure 4e,g). These short axon phenotypes of *shot* seemed to mirror the occurrence of MT disorganization in *shot* mutant neurons, where axonal bundles lost their parallel arrangements and took on curled, criss‐crossing appearances (referred to as MT curling; Figure 4c). Like the axon length phenotype, axonal MT curling was not influenced by LatA treatment or loss of Chic (Figure 4e,g,h), thus demonstrating a further parallel between both phenotypes.

### 2.5 Shot seems to work through two redundant mechanisms in MT bundle maintenance

**FIGURE 5:**
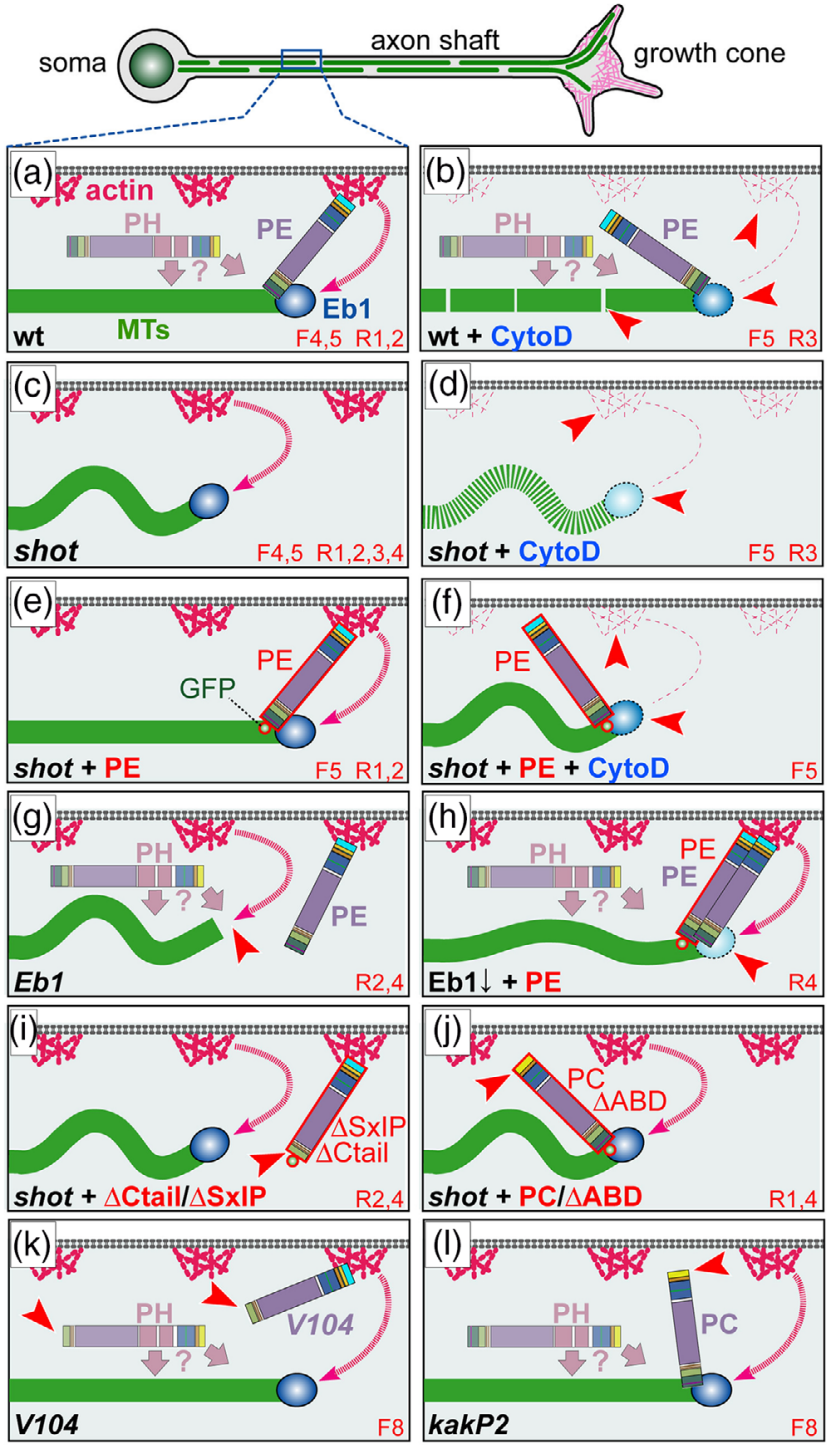
Schematic overview of existing experiments addressing Shot roles in microtubule (MT) bundle organization. (a) Schematic section of the axonal surface including cortical actin (magenta) anchoring the Shot N‐terminus of CH1‐containing isoforms (here PE) and promotes MT polymerization (dashed magenta arrow; Qu et al., 2017); via its C‐terminus, Shot‐PE binds EB1 (dark blue) and MTs (green) thus cross‐linking polymerizing MT tips to the cortex and guiding their extension into parallel bundles (Alves‐Silva et al., 2021); the plakin repeat region (PRR)‐containing PH isoforms (shown in pale) does not bind F‐actin but we propose that it contributes to MT bundle formation/maintenance through yet unknown mechanisms (‘‘?’’; see Discussion). (b–l) Different experimental conditions and their impact on MT behaviors; red numbers at bottom right indicate the information source: ‘‘F’’ refers to figure numbers in this publication, ‘‘R’’ indicates external references: (R1) (Sánchez‐Soriano et al., 2009), (R2) (Alves‐Silva et al., 2012), (R3) (Qu et al., 2017), (R4) (Hahn et al., 2021); red arrow heads point at specific functional lesions in the different conditions. Explanations: in wild‐type neurons, CytoD eliminates cortical actin and weakens MT polymerization (pale Eb1 with dashed outline), not strong enough to affect parallel MT arrangements but leading to MT gaps (interrupted green line; b); in the absence of Shot, MTs curl (c) and MT networks shrink (they become vulnerable to lack of actin‐promoting effects causing more severe loss of MTs; stippled green line; d); guiding function is fully re‐instated by targeted expression of Shot‐PE (e; expression constructs red encircled with a green GFP dot at their ends); Shot‐PE fails to guide MTs in the absence of actin, but it protects MT polymerization (f); Eb1 deficiency eliminates the F‐actin/MT/Eb1 guidance mechanism, and might even be involved in the alternative mechanism of Shot (arrow of pale PH toward MT plus end; g; see Discussion); MT curling upon reduced Eb1 levels (Eb1↓) can be rescued with Shot‐PE expression (h); MT curling caused by loss of Shot (or Eb1; see Ref. 4) cannot be rescued with Shot‐PE variants that lack Ctail or Eb1‐binding SxIP motifs (i; see Figure 7c) or the CH1 domain (j); absence of the same domains in *shot^V104^* (k) or *shot^kakP2^* (l) does not cause MT curling. We propose that the presence of the Shot‐PH isoform (faintly shown in a, b, k, and l) protects axons against loss of actin or F‐actin/MT/Eb1 guidance mechanism, that is, conditions which cause severe curling in the other experimental settings (c, f, g, i, and j)

Previous work had demonstrated that Shot prevents MT curling through a guidance mechanism involving F‐actin/Eb1/MT cross‐linkage: via its N‐terminus Shot binds cortical F‐actin and via its C‐terminus to MTs and Eb1—thus guiding the extension of polymerizing MTs along the axonal cortex into parallel bundles; this F‐actin/Eb1/MT guidance mechanism is supported by numerous structure‐function, loss‐of‐function, and pharmacological and genetic interaction studies (details in Figure 5; Alves‐Silva et al., 2012; Hahn et al., 2021; Qu et al., 2019; Sánchez‐Soriano et al., 2009).

The F‐actin/Eb1/MT guidance mechanisms would predict that removal of cortical F‐actin from wild‐type neurons (which can be achieved with the F‐actin‐inhibiting drug CytoD, but less so with LatA or loss of Chic; Qu et al., 2017) should mimic the *shot* mutant MT curling phenotype. However, CytoD application to wild‐type neurons failed to cause MT curling; instead it caused a deficit in MT polymerization leading to gaps in MT bundles (likely due to loss or shortening of MTs, which therefore fail to overlap; Figures S1b, S6b, and S5b; Qu et al., 2017)—which may also explain why loop suppression upon CytoD application (Figure S1) does not enhance axon growth as observed with LatA (Sánchez‐Soriano et al., 2010).

**FIGURE 6:**
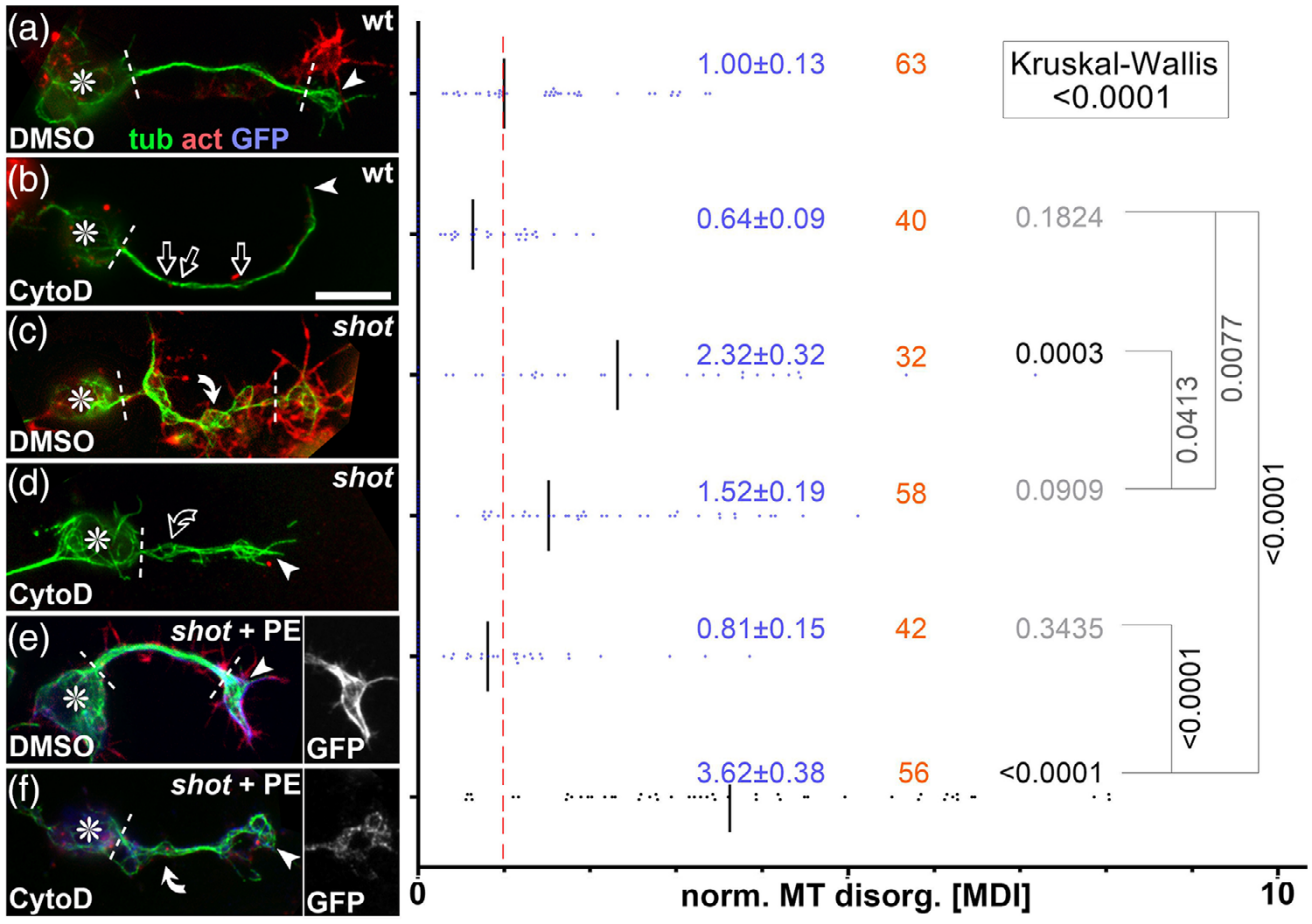
Cytochalasin D (CytoD) experiments confirming the F‐actin‐dependent guidance mechanism of Shot. Left side: Primary neurons of different genotypes (as indicated: wt, wild‐type; *shot*, *shot^3/3^*; *shot* + PE, *shot^3/3^* expressing Shot‐PE) at 6–8 h in vitro (HIV) on ConA, treated with vehicle (DMSO) or CytoD as indicated, and stained for tubulin (green), actin (red), or GFP (blue); asterisks indicate cell bodies, arrowheads the tip of axons, white dashed lines demarcate the axon shaft, open arrows gaps in axonal tubulin bundles, and white/open curved arrows areas of normal/fractured microtubule (MT) curling; scale bar in (b) represents 10 μm in all images. Right side: Quantification of the degree of MT curling in the axon shafts (between white dashed lines or dashed line and arrowhead in images on the left) of each genotype, measured in MT disorganization index (MDI) and normalized to wildtype controls (red dashed line); numbers of neurons analyzed are indicated in orange, mean ± SEM in blue and results of Mann–Whitney rank sum tests are shown in black/gray. Further explanations are given in Figure 5

The fact that CytoD failed to mimic the MT curling phenotype of *shot* mutant neurons (Figure 6b vs. c) might indicate that F‐actin/Eb1/MT guidance is not the only mechanism through which Shot contributes to MT bundle maintenance. For example, Shot might work through further isoforms beyond Shot‐PE (the only isoform shown so far mediating MT bundle maintenance; Figure 5e,f,h–j). To test this possibility, we used Shot‐deficient mutant neurons in which the MT curling phenotype was rescued by Shot‐PE::GFP so that Shot‐PE was the sole isoform present in these neurons (Figures 5e and 6e). These neurons were normal in appearance. However, when treated with CytoD, strong MT curling was induced (Figures 6f and 5f). This indicated that Shot‐PE‐mediated F‐actin/Eb1/MT guidance is not sufficient when F‐actin is removed from axon shafts, and might suggest that these neurons lack some additional bundle‐maintaining functions of Shot that are actin‐independent.

**FIGURE 7:**
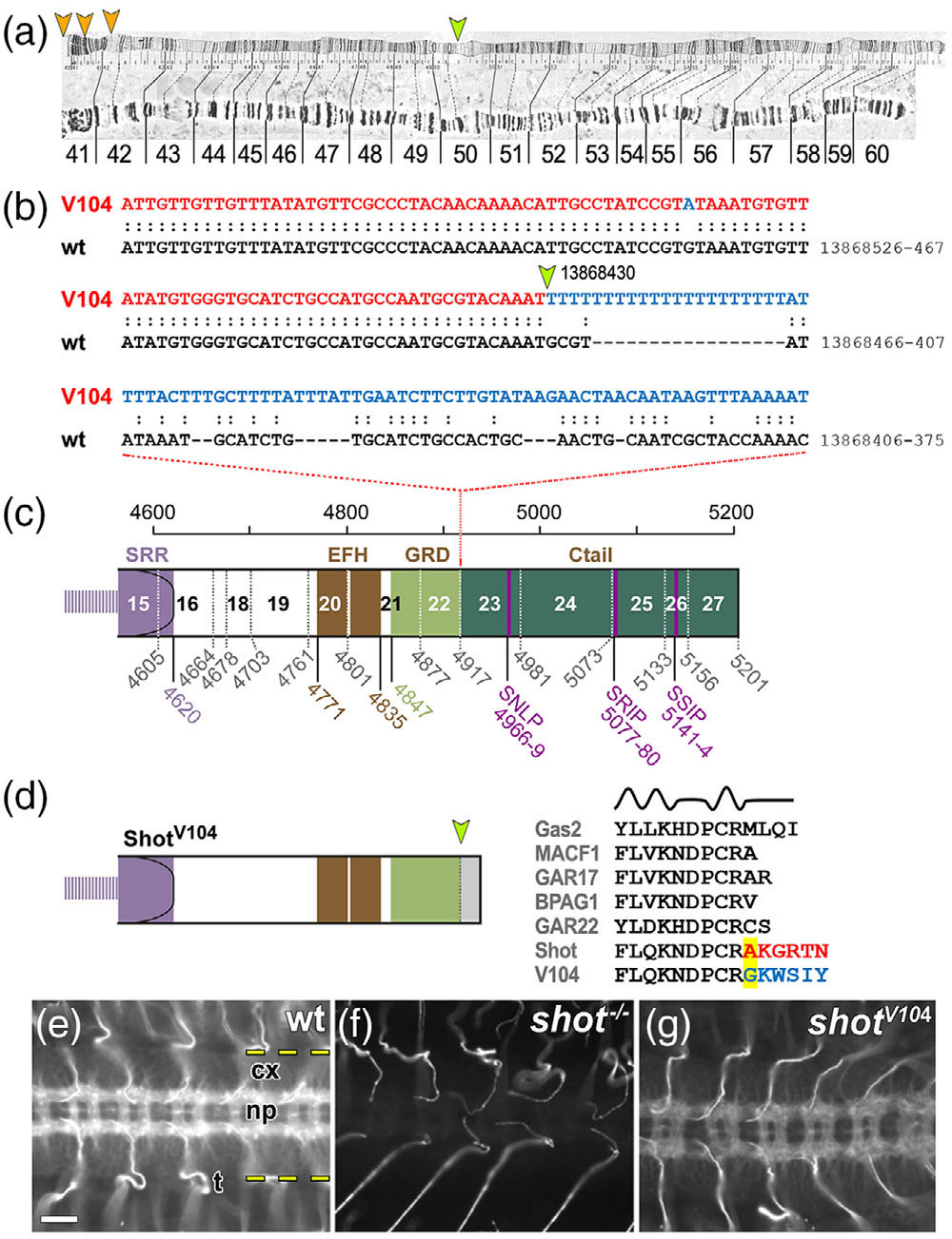
The *shot^V104^* breakpoint removes the Ctail. (a) View of the 2R polytene chromosome (Lindsley & Zimm, 1992) indicating the mapped breakpoint in 50C (orange arrow) and potential sites of the second breakpoint in the centromeric region of 2R (orange arrowheads) suggested by the mapping positions of several clones with matching sequences (when using the BLAST function in flybase.org and the blue sequence in (b) as query); clones with matching sequences: *DS03708* (42A4‐42A5), *BACR04E10* (41C‐41D), *BACR07J16* (41C‐41C), *BACR05A24* (41C‐41D), *BACR05A24* (41C‐41D), and *BACR03D04* (40D‐40D). (b) Alignment of the wild‐type and *V104* mutant genomic sequences of *shot* indicating the breakpoint (yellow arrow) in position 13,868,412 (primary assembly 2R: 13,864,237‐13,925,503 reverse strand) and the newly fused sequence in *shot^V104^* (blue) likely derived from the other end of the inversion that would usually be situated near the position of the second breakpoint (orange arrows in a). (c) Schematic of the Shot‐PE protein (FBtr0087618) drawn to scale and indicating domain/motif borders (colored numbers below; compare Figure 1a) as well as exon borders (stippled vertical lines, gray numbers, exon numbers indicated between lines); the *V104* breakpoint is situated in intron 22/23. (d) The predicted V104 protein is truncated behind the GRD (yellow arrow) potentially reading into intronic sequences (gray). Comparison of the V104 sequence at the breakpoint (highlighted yellow) with sequences of GRDs from normal Shot and other GRD‐containing proteins (listed in gray; taken from Alves‐Silva et al., 2012) strongly suggest that the truncation does not affect the final α‐helix and amino acid changes occur behind the GRD. (e–g) Ventral nerve cords of stage 16 embryos (cx, cortex containing cell bodies; np, neuropile containing synapses and as‐/descending tracts; dashed yellow lines demarcate outlines of the ventral nerve cord; t, trachea) stained with the Shot‐C antibody against the C‐terminal part of the spectrin repeat rod (Figure 1a; Strumpf & Volk, 1998); staining reveals the presence of protein in wild‐type (e), absence in homozygous *shot* null mutant embryos (f) and presence in hemizygous *shot^V104/MK1^* mutant embryos where reduced expression is due to the absence of one gene copy (*V104* is over the *MK1* deficiency); scale bar in E represents 20 μm in (E–G)

In contrast to Shot‐PE::GFP, the Shot‐PC::GFP or Shot‐PE‐ΔCtail::GFP variants failed to rescue MT curling in *shot* mutant neurons (Figure 5i,j), arguing that the CH1 and Ctail domains of Shot are essential for F‐actin/Eb1/MT guidance. In *shot^kakP2^* and *shot^V104^* mutant animals, these two domains are specifically missing from all isoforms of endogenous Shot proteins (details in Figures 1a and 7; Bottenberg et al., 2009; Gregory & Brown, 1998). Therefore, these two alleles are expected to eliminate the endogenous F‐actin/Eb1/MT guidance function but potentially leave other functions of Shot intact (‘‘PH’’ in Figure 5k,l).

**FIGURE 8:**
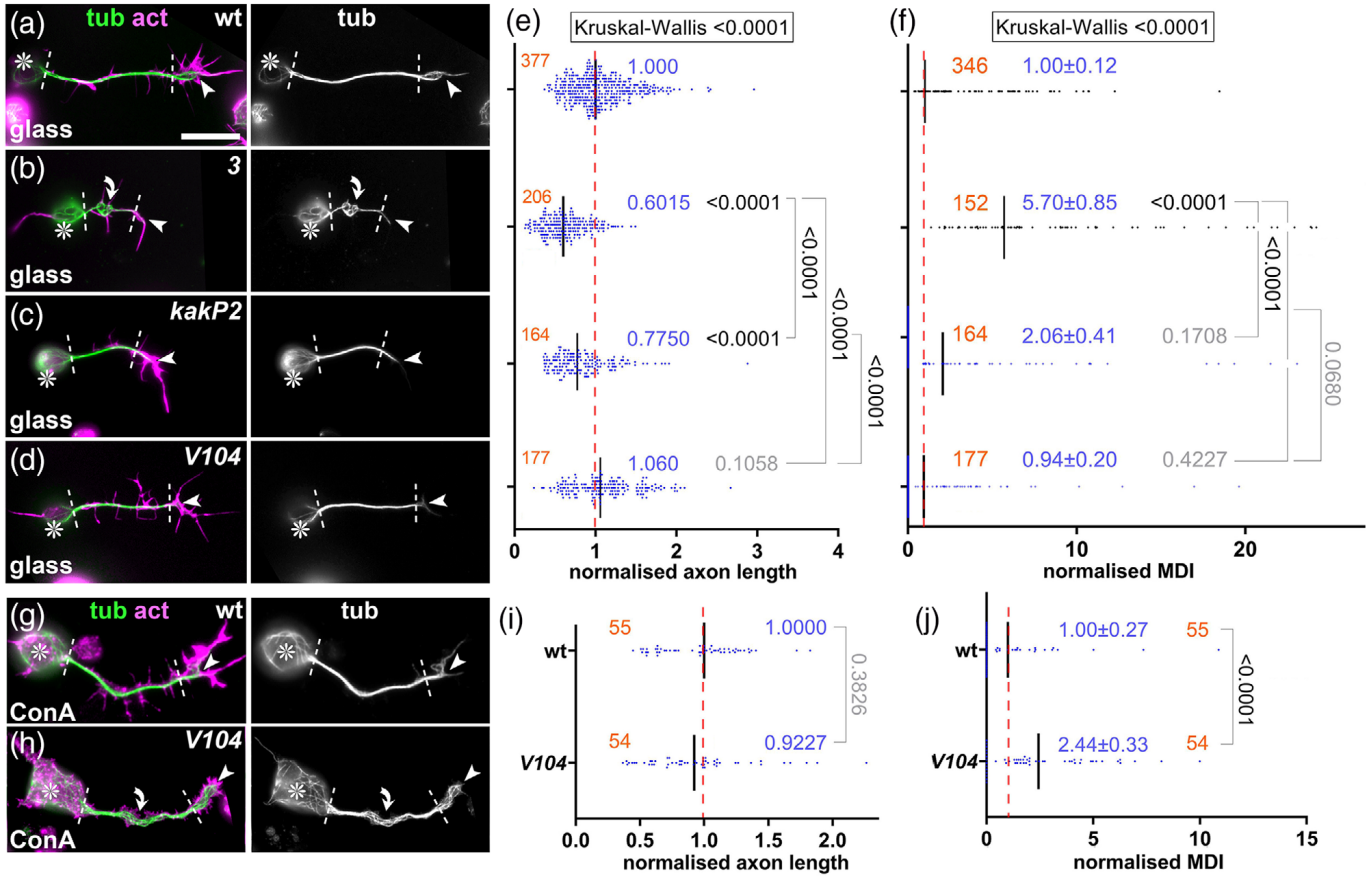
Phenotypes of *shot^kakP2^* and *shot^V104^* mutant primary neurons. (a–d, g, h) Images of neurons at 6–8 h in vitro (HIV) of different genotypes (wt, wild‐type; 3, *shot^3/3^*; *kakP2*, *shot^kakP2/kakP2^*; *V104*, *shot^V104/Df(MK1)^*) cultured on glass (a–d) or ConA (g and h) and stained for tubulin (green), actin (magenta) or GFP (blue); grayscale images on the right show only the tubulin channel; asterisks indicate cell bodies, arrowheads the tips of axons, dashed white lines demarcate axon shafts, curved arrows areas of MT curling; scale bar in (a) represents 20 μm in (a–d) and 10 μm in (g) and (h). (e, f, i, and j) Quantifications of axon length (e and i) and microtubule (MT) curling (measured in MT disorganization index [MDI]; f and j), both normalized to wild‐type controls (red dashed line); numbers of neurons analyzed are indicated in orange, mean ± SEM in blue and results of Mann–Whitney rank sum tests are shown in black/gray

When analyzed in whole embryos, both mutant alleles clearly caused hypomorphic loss‐of‐function mutant phenotypes: *shot^kakP2^* strongly affected the nervous system (Bottenberg et al., 2009; Gregory & Brown, 1998), whereas *shot^V104^* defects seemed to restrict to non‐neuronal tissues (Figure S6). We next cultured primary neurons from these embryos and measured the degree of MT curling in the axon shaft, which is the area where the guidance mechanism is expected to make its prime contributions. In *shot* ^3^ null mutant neurons used as positive controls, severe MT curling occurred along axon shafts; in contrast, *shot^V104^* mutant neurons showed no obvious phenotypes, and *shot^kakP2^* revealed only a trend toward curling (Figure 8a–d,f). For *shot^V104^* mutant neurons, we repeated the experiment, but this time culturing them on concanavalin A which is a more challenging condition causing greater mechanical strain (Prokop et al., 2012). When challenged this way, *shot^V104^* mutant neurons displayed robust MT curling. This suggests that loss of the F‐actin/MT/Eb1 guidance mechanism weakens the overall machinery of MT bundle maintenance: under modest conditions, its absence can be masked by other bundle‐maintaining functions of Shot, but this becomes insufficient when the mechanical challenge is increased.

## 3 DISCUSSION

### 3.1 Roles of Shot in axonal MT regulation involve various isoform‐specific actin‐dependent and ‐independent functions

Spectraplakins are well conserved across the animal kingdom; they are essential cytoskeletal regulators in neurons, linked to severe MT curling in mammals and *Drosophila* alike (Dalpe et al., 1998; Eyer et al., 1998; Sánchez‐Soriano et al., 2009; Voelzmann et al., 2017). Many mechanistic insights were gained using *Drosophila* Shot as a model, and F‐actin/Eb1/MT guidance has emerged as a central theme that is consistent also with roles in non‐neuronal cells (Kodama et al., 2003; Ricolo & Araujo, 2020). Here, we refined our understanding of Shot's actin dependency during MT regulation, while also proposing the co‐existence of actin‐independent functions involved in MT bundle promotion.

### 3.2 Shot's roles in spool formation are regulated by F‐actin

Our findings suggest that F‐actin is an important instructor of Shot's MT‐regulating roles. For example in GCs, Shot is an essential regulator of spool formation in an F‐actin‐dependent manner: (1) spools can be suppressed when depleting F‐actin (LatA, CytoD; Figures 2d and S1b; Sánchez‐Soriano et al., 2010), (2) when changing the properties of F‐actin networks (CK666; Figure 2D), or (3) when changing Shot's actin‐binding properties as observed with Shot‐PC, Shot‐PE‐ΔABD, Shot‐PE‐ΔCH2, Shot‐PE‐Moe, and Shot‐PE‐Life (Figure 1l). In contrast, F‐actin networks of the axon shaft are far less prominent (Xu et al., 2013), which seems sufficient for F‐actin/Eb1/MT guidance but not enough to induce prominent changes to MT bundles even when overexpressing Shot‐PE. In contrast, Shot‐PE‐Life was able to induce abnormal MT bundle split in the shaft (Figures 3 and S4), suggesting that increased F‐actin affinity is sufficient to tip the balance in an F‐actin‐sparse environment and change the MT‐regulating behavior of Shot.

Taken together, these experiments suggest that proper Shot function requires well‐balanced interaction with F‐actin networks, and the spectacular phenotypes we observe with Shot‐PE‐Life (Figure 3 and Figure S4) suggest that our findings can be turned into new genetic tools to investigate how changes in cytoskeletal organization may impact on neuronal architecture, dynamics, and even physiology.

Our experiments with Shot‐PE‐Life have demonstrated a clear F‐actin‐dependence of the induced MT phenotypes (Figure 3f,g). They also suggested that this construct was able to induce ectopic F‐actin in axon shafts (Figure 3 and Figure S4), potentially reflecting mutual regulation mediated through Shot. This may involve known roles of the Shot C‐terminus in promoting F‐actin nucleation (Sánchez‐Soriano et al., 2009): for example, the strong localization of Shot‐PE‐Life along axon shafts might trigger a positive feedback loop by nucleating more F‐actin which then enhances Shot‐PE‐Life localization.

In normal Shot‐PE, direct binding through the CH domains does not appear sufficient to trigger changes in spool‐inducing functions of Shot, and also the plakin domain appears functionally involved. To our knowledge, the only plakin domain‐binding factors reported so far are transmembrane adhesion factors including integrins and collagen XVII at mammalian hemidesmosomes (Aumailley et al., 2006) and potentially the N‐CAM homologue Fasciclin II in *Drosophila* neurons (Voelzmann et al., 2017). Since the localization of such adhesion factors is dependent on F‐actin (Woichansky et al., 2016), they might provide a potential second route through which F‐actin can influence Shot activity.

In summary, we have built a case for regulatory impacts of F‐actin networks on Shot function which, in turn, trigger MT network changes that impact on axon growth; this is best exemplified by the negative correlation between spool formation and axon growth (Figure 4a; Dent et al., 1999; Sánchez‐Soriano et al., 2010).

### 3.3 Shot displays prominent F‐actin‐independent roles in axons

Shot also plays major roles in maintaining MT bundles in axon shafts. We confirmed here the importance of F‐actin/Eb1/MT guidance for parallel bundle arrangements (Alves‐Silva et al., 2012; Hahn et al., 2021; Figures 5, 6, and 8). We believe that roles of F‐actin in this context are merely permissive with little regulatory potential because F‐actin networks in axon shafts appear sparse and far less dynamic compared to GCs. However, these permissive roles are nevertheless important as clearly demonstrated by MT curling in *shot* mutant neurons which correlates with drastic growth reduction. We suggest that curling in the axon shaft diverts MT polymerization away from the axon tip, thus reducing their likelihood of reaching the GCs and contribute to axon growth events.

In addition to F‐actin/Eb1/MT guidance, we also presented strong arguments for additional functions of Shot in MT bundle maintenance that are independent of this form of cross‐linkage (Figure 5b,k,l). Considering the enormous importance that MT bundles have for the long‐term survival of axons (Hahn et al., 2019; Prokop, 2020), it would make biological sense to have redundant mechanisms to maintain these bundles and prevent axonopathies (Prokop, 2021).

In our view, the best candidate to mediate F‐actin‐independent functions of Shot is the unique Shot‐PH isoform. Shot‐PH is highly expressed in the nervous system, has a C*‐type N‐terminus (non‐F‐actin‐binding like Shot‐PC; Figure 1a), and stands out as the only isoform containing a large central PRR (Figure S7b; flybase.org reference: FBgn0013733; Hahn et al., 2016; Röper & Brown, 2003; Voelzmann et al., 2017).

PRRs are conserved in mammalian dystonin and ACF7/MACF1 (Voelzmann et al., 2017), but very little is known about their role or potential binding partners. The PRR of *Drosophila* Shot plays regulatory roles at epithelial adherens junctions through unknown mechanisms (Röper & Brown, 2003). In mammals, the PRR‐containing isoform MACF1b was shown to associate with the Golgi (Lin et al., 2005). However, we struggle to imagine mechanisms through which Golgi‐related mechanisms in the cell body could maintain MT bundles in the axon under conditions where F‐actin/Eb1/MT guidance is abolished. In our view, investigating the potential roles and mechanisms of PRRs in axons would therefore have great potential to deliver new mechanisms that can advance our understanding of axon maintenance and architecture (Prokop, 2020).

As a first step to study PRRs, we generated flies carrying a CRISPR/Cas9‐mediated PRR deletion. Unfortunately, *shot^ΔPRR^* mutant flies displayed unexpected splicing defects resulting in a strong loss‐of‐function mutant allele (details in Figure S7); while being potentially interesting for molecular geneticists that work on splicing mechanisms, this allele was unsuitable for our purposes. An alternative strategy could be to identify PRR‐binding or PRR‐associating proteins (Lin et al., 2021), and then use versatile *Drosophila* genetics in combination with our culture model (Prokop et al., 2013) to establish their potential involvement in bundle maintenance.

Among the PRR‐interacting proteins, we would expect to find also Eb1‐binding proteins or even Eb1 itself (note that PRR contains a potentially Eb1‐interacting SNLP motif as similarly found in the Ctail; Figure 7c; Honnappa et al., 2009). A link from the PRR to Eb1 could explain an important conundrum posed by the current data: loss of Eb1 causes MT curling, but the deletion of the Eb1‐binding Ctail from all Shot isoforms does not (*shot^V104^*; Figures 5g vs. 5k, 7, and 8d–f)—the PRR might be the missing puzzle piece establishing alternative links to Eb1.

Taken together, we propose a system of redundant Shot‐mediated mechanisms that promote axonal MT bundle architecture—in addition to other factors expected to be involved, such as classical MAPs or mitotic kinesins (Guha et al., 2021; Hahn et al., 2019; Prokop, 2020). Such robust redundancy makes sense when considering the enormous importance of these MT bundles for axonal longevity (Prokop, 2021). We believe that the study of Shot‐PH can establish new investigative paths toward a more profound understanding of axon architecture, thus bridging a gap in the field that may provide important explanations for a wide range of axonopathies and new avenues for their treatment.

## 4 MATERIALS AND METHODS

### 4.1 Fly strains

The following fly stocks were used: Oregon R as wild‐type control and the strong loss‐of‐function or null alleles *chic^221^* (Verheyen & Cooley, 1994)*, shot^3^* (Kolodziej et al., 1995), *shot^kakP2^* (synonymous to *P{lacW}shotk03405*; Gregory & Brown, 1998), *shot^HG25^* (Prokop et al., 1998), and *shot^V104^* (Strumpf & Volk, 1998). All mutant stocks were kept and selected with *twi‐Gal4/UAS‐GFP* green balancers (Halfon et al., 2002). Existing transgenic lines we used included the *scabrous‐Gal4*, *eve‐Gal4^RN2E^* and *stripe‐Gal4* driver lines (Fujioka et al., 1999; Mlodzik et al., 1990; Subramanian et al., 2003), *UAS‐mCD8‐GFP* (Luo et al., 1994), *UAS‐shot‐RE‐GFP*, and *UAS‐shot‐RC‐GFP* (Bloomington Stock Center #29044 and #29042, respectively; Lee & Kolodziej, 2002), *UAS‐EGC‐GFP* (Subramanian et al., 2003), *UAS‐shot‐RE‐Δplakin‐GFP* (Bloomington Stock Center #29649; Bottenberg et al., 2009), and *UAS‐Act5C‐GFP* (Bloomington Stock Center #7309; Kelso et al., 2002).

### 4.2 *Drosophila* primary neuronal cell culture

Neuronal cell cultures were generated as detailed elsewhere (Prokop et al., 2012; Voelzmann & Sánchez‐Soriano, 2021). Embryos were dechorionated for 1.5 min in 50% domestic bleach, correct stages (usually stage 11; Campos‐Ortega & Hartenstein, 1997), and genotypes were selected under a fluorescent dissecting microscope, transferred to sterilized centrifuge tubes containing 100 μl of 70% ethanol, washed in sterile Schneider's medium containing 20% fetal calf serum (Schneider's/FCS; Gibco), and, eventually, homogenized with micropestles in 1.5 ml centrifuge tubes containing 21 embryos per 100 μl dispersion medium (Prokop et al., 2012). They were left to incubate for 4 min at 37°C. Dispersion was stopped with 200 μl Schneider's/FCS, cells were spun down for 4 min at 650 g, supernatant was removed, and cells were resuspended in 90 μl of Schneider's/FCS; 30 μl drops were placed in culture chambers and covered with cover slips. Cells were allowed to adhere to cover slips for 90–120 min either directly on glass or on cover slips coated with a 5 μg/ml solution of concanavalin A, and then grown as a hanging drop culture at 26°C usually for 6–8 h.

Transfection of *Drosophila* primary neurons was executed as described previously (Qu et al., 2019). In brief, 70–75 embryos per 100 μl dispersion medium were used. After the washing step and centrifugation, cells were resuspended in 100 μl transfection medium (final media containing 0.1–0.5 μg DNA and 2 μl Lipofectamine 2000 [L2000; Invitrogen]), incubated following manufacturer's protocols (Thermo Fisher, Invitrogen) and kept for 24 h at 26°C. Cells were then treated again with dispersion medium, resuspended in culture medium and plated out as described above.

### 4.3 Drug application and immunohistochemistry

For drug treatments, solutions were prepared in cell culture medium from stock solutions in DMSO. Cells were treated for 4 h with 200 nM latrunculin A (Biomol International), 0.4 μg/ml cytochalasin D (Sigma), or 100 nM CK666 (Sigma), respectively. For controls, equivalent concentrations of DMSO were diluted in Schneider's medium.

Culture medium was carefully removed and cells fixed for 30 min with 4% paraformaldehyde in 0.05 M phosphate buffer (pH 7–7.2), then washed in phosphate buffered saline with 0.3% TritonX‐100 (PBT). Incubation with antibodies was performed in PBT without blocking reagents. The following antibodies were used: anti‐α‐tubulin (clone DM 1A, 1:1000, mouse, Sigma), anti‐Shot‐C raised against aa3450‐4714 (C‐terminal end of the spectrin repeat region; Figure 1a; guinea pig; 1:200; Strumpf & Volk, 1998); anti‐GFP (1:500, goat, Abcam), and FITC‐, Cy3‐, or Cy5‐conjugated secondary antibodies (1:200, purified from donkey, Jackson Immunoresearch). F‐actin was stained with TRITC‐ or Cy5‐conjugated Phalloidin (Sigma; 1:100). Coverslips with stained neurons were mounted on slides using Vectashield medium (Vector labs) or ProLong Gold Antifade Mountant (ThermoFisher Scientific).

### 4.4 Stage 17 embryo dissections

Dissection of late stage 17 embryos (stages according to Campos‐Ortega & Hartenstein, 1997) was carried out as described in great detail elsewhere (Budnik et al., 2006). In brief, embryos were dissected flat in PBS on Sylgard‐coated cover slips with the help of sharpened tungsten needles and Histoacryl glue (Braun, Melsungen, Germany), followed by 1 h fixation in 4% paraformaldehyde, 1 h wash in PBT, and the same histochemical staining steps as mentioned above using the following antibodies: anti‐FasII (1D4 2F3, DSHB; mouse, 1:20; Van Vactor et al., 1993), anti‐GFP (see above), and anti‐Synaptotagmin (rabbit polyclonal; 1:1,000; Littleton et al., 1993). Embryos were cut out from the glue using razor blade splinters or the tungsten needles and embedded in glycerol.

### 4.5 Imaging and image analysis

Standard imaging was performed with AxioCam 506 monochrome (Carl Zeiss Ltd.) or MatrixVision mvBlueFox3‐M2 2124G digital cameras mounted on BX50WI or BX51 Olympus compound fluorescent microscopes. Measurements from images were carried out in the fixed preparations using ImageJ (segmented line and freehand selection tools). Only neurites at least twice the length of the soma diameter were analyzed using α‐tubulin staining and measuring from the edge of the cell body to the tips of the axons (excluding MTs in filopodia); in cases where neurites branched, the longer branch was measured, in cases where two neurites extended from a single cell, the longer value was taken. The degree of disorganized MT curling in axon shafts was established either as binary readout (% of neurons with disorganization) or as “MT disorganization index” (MDI) described previously (Qu et al., 2019, 2017); in short: the area of disorganized curling was measured with the freehand selection tool in ImageJ; this value was then divided by axon length (see above) multiplied by 0.5 μm (typical axon diameter, thus approximating the expected area of the axon if it were properly bundled); in this study, MDI measurements were restricted to the axon shaft, that is, from the cell body to the base of GCs (white dashed lines in Figures 6 and 8). Filopodia numbers were counted per neurite. GCs containing looped MT bundles (spools) were classified according to previous publications (Sánchez‐Soriano et al., 2010). Graphpad Prism was used to describe data, perform statistical tests, and generate final graphs. Data were usually not normally distributed, and the median was determined for axon length; since MDI measurements contain many zero‐value data, the mean and standard error of the mean (SEM) had to be used to obtain meaningful numbers. For statistical analyses, the chi‐square test was used when comparing percentages, Kruskal–Wallis one‐way analysis of variance (ANOVA) test to compare groups, and Mann–Whitney rank sum tests (indicated as *p*_MW_) to compare pairs of data. For the correlation, *r*‐ and *p*‐values were determined via nonparametric Spearman correlation analysis (tests showed that data are not distributed normally). The data used for our analyses will be made available on request from the authors.

### 4.6 Electron microscopy

Procedures followed protocols published in detail elsewhere (Budnik et al., 2006). In brief, embryos were injected with 5% glutaraldehyde in 0.05 M phosphate buffer, pH 7.2, the injected specimens were cut open at their tips with a razor blade splinter, postfixed for 30–60 min in 2.5% glutaraldehyde in 0.05 M phosphate buffer, briefly washed in 0.05 M phosphate buffer, fixed for 1 h in aqueous 1% osmium solution, briefly washed in dH_2_O, treated en bloc with an aqueous 2% solution of uranyl acetate for 30 min, dehydrated, and then transferred to araldite or TAAB LV (TAAB Laboratories Equipment, Berkshire, UK). Serial sections of 30–50 nm (silver‐gray) thickness were transferred to formvar‐covered carbon‐coated slot grids, poststained with lead citrate for 5–10 min, and then examined on a JEOL 200CX (Peabody, MA, USA) or Hitachi H600 (Tokyo, Japan).

### 4.7 Cloning of *shot* constructs

All primers used for the cloning steps are listed in Table 1. The N‐terminal CH deletions (ΔCH1, ΔCH2, ΔABD) were made by PCR amplification of two DNA fragments flanking the CH domains, using respective primers listed in the table which contained homologous sequences to anneal them into a template for further PCR amplification. The PCR product was digested and ligated into *pET20b* vector (Novagen) using AscI and XhoI. To insert alternative actin‐binding domains (Lifeact source: *pCMVLifeAct‐TagGFP2* vector, Ibidi; Moesin was a gift from Tom Millard; Millard & Martin, 2008), they were amplified in parallel to the two CH domain‐flanking sequences and annealed in triplet constellation for making the template. Polymerase chain reaction (PCR) amplification was used to add NotI/XbaI restriction sites to the 5′ and 3′ ends followed by digestion and ligation into a modified version of the *pUASp* vector (Invitrogen; kindly provided by Tom Millard) which confers ampicillin resistance and tags the construct N‐terminally with eGFP (referred to as *pUASp‐eGFP*). N‐terminal constructs in *pUASp‐eGFP* were amplified in chemically competent TOP10 *Escherichia coli* and used for transfection into primary neurons (see above).

**TABLE 1:** List of primers

Name	Sequence
pUASP_Nterm_Fw	TTAATCGCGGCCGCAATGGCATCGCATTCCTAC
pUASP_Nterm_Rev	GGCAACTCTAGACTAAAGGATAACCTCGCGATC
pUASP_Nterm_seq_Fw	GACAACCACTACCTGAGC
pUASP_Nterm_seq_Rev	CTTGACCATGGGTTTAGG
Nterm_ΔCH1_Fw_3b	CTCACCCAGTTTAAAGACGAACGCATCTCCGATATTGTTGTGGGCAAAGAG
Nterm_ΔCH1_Rev_3a	CTCTTTGCCCACAACAATATCGGAGATGCGTTCGTCTTTAAACTGGGTGAG
Nterm_ΔCH2_Fw_2b	GATATTGTTGTGGGCAAAGAGGACGAGCCACCCTCTATCCATCCACTC
Nterm_ΔCH2_Rev_2a	GAGTGGATGGATAGAGGGTGGCTCGTCCTCTTTGCCCACAACAATATC
Nterm_ΔCH_Fw_4b	CTCACCCAGTTTAAAGACGAACGCGAGCCACCCTCTATCCATCCACTC
Nterm_ΔCH_Rev_4a	GAGTGGATGGATAGAGGGTGGCTCGCGTTCGTCTTTAAACTGGGTGAG
Nterm_lifeact_Fw_6b	GATTTGATCAGAAATTCGAAAGCATCTCAAAGGAAGAAGAGCCACCCTCTATCCATCCACTC
Nterm_lifeact_Rev_6a	GATGCTTTCGAATTTCTTGATCAAATCTGCGACACCCATGCGTTCGTCTTTAAACTGGGTGAG
Nterm_moesin_Fw_7b	CGCGTCGATCAGTTTGAGAACATGGAGCCACCCTCTATCCATCCACTC
Nterm_moesin_Rev_7a	CTGGCGAACGTTCTCGCGATGAATGGCATCGCGTTCGTCTTTAAACTG
Nterm_moesin_Fw_7c	CAGTTTAAAGACGAACGCGATGCCATTCATCGCGAGAACGTTCGCCAG
Nterm_moesin_Rev_7c	GAGTGGATGGATAGAGGGTGGCTCCATGTTCTCAAACTGATCGACGCG
Nterm_Seq_Fw_New	CCACAACGGTTTCCCTCTAG
Nterm_seq_Rev_New	GCTAGTTATTGCTCAGCG
Nterm_Recomb_Fw	GAGAACAGCAGCAGTCCG
Nterm_Recomb_Rev	CAGGTAGGCGGTCTTCTC

For making the respective full‐length Shot‐PE constructs carrying the N‐terminal variations (*UAS‐Shot‐PE‐ΔABD‐GFP*, now available at Bloomington Stock Center: #93282; *UAS‐Shot‐PE‐ΔCH1‐GFP*; *UAS‐Shot‐PE‐ΔCH2‐GFP*; *UAS‐Shot‐PE‐Life‐GFP*, now available at Bloomington Stock Center: #93283; *UAS‐Shot‐PE‐Moe‐GFP*; *UAS‐Shot‐PE‐ΔABD‐GFP*), *Nterm_Recomb* primers were used to amplify the N‐terminal constructs from the *pET20b* vector. These were then used to replace the *GalK* cassette in full‐length shot‐RE within *M‐6‐attB‐UAS‐1‐3‐4* vector via recombineering strategies (Alves‐Silva et al., 2012) and the positive/negative selection strategy (Warming et al., 2005). The *GalK* cassette was originally inserted into *M‐6‐attB‐UAS‐1‐3‐4 shot‐RE*‐borne *shot‐RE* by using similar recombineering steps with *GalK* which had been amplified with primers that added the same homology arms as mentioned above.

The completed constructs in *M‐6‐attB‐UAS‐1‐3‐4* vector were amplified in Epi300 competent cells (EpiCentre) in LB‐Chloramphenicol medium, adding CopyControl solution (EpiCentre) 2 h before the miniprep. Amplified constructs were used to generate transgenic flies (outsourced to BestGene, Chino Hills, CA 91709, US) using PhiC31‐mediated site‐specific insertion using a specific attB landing site on the third chromosome (*PBac{y* ^+^ *‐attP‐3B}CG13800^VK00031^*; Bloomington Stock Center #9748; Alves‐Silva et al., 2012). This same landing site was used for all constructs to avoid position effects and achieve equal expression levels of all constructs (Bischof et al., 2007).

### 4.8 Generating *shot^ΔPRR^* mutant flies

The PRR domain (exon 12 of shot‐RH, FBtr0087621) was excised from the *shot* genomic region and replaced with 3xP3‐DsRed (driving DsRed expression in the eye) via CRISPR/Cas9‐mediated homology‐directed repair. Suitable gRNA target sites (5′ gRNA: GAGTGCTAACCTCCTGACTAG, 3′ gRNA: CTGTTCTGCCGGCAGGAGCAC) were identified by CRISPR optimal target finder (Gratz et al., 2014) and cloned into pCFD4‐U6:1_U6:3tandemgRNAs (gift from Simon Bullock; Addgene plasmid #49411; RRID:Addgene 49411) via Gibson assembly (NEB). Adjacent 2 kb 5′ and 3′ homology regions were cloned into *pHD‐DsRed‐attP* (gift from Melissa Harrison, Kate O'Connor‐Giles, and Jill Wildonger, Addgene plasmid #51019, RRID:Addgene_51019) 5′ region via EcoRI/NotI, 3′ region via BglII/PstI) using the following primer pairs:

- 5′ HR fwEcoRI: AAAAGAATTCctcgtttgttcgctcttaccc.
- 5′ HR revNotI: AAAAGCGGCCGCCTGAAAGGATTCGATTAGAACTTTATTAG.
- 3′ HR fwBglII AAAAAGATCTGTAAGTCTCAGAACACTCGAGG.
- 3′ HR revPstI AAAACTGCAGTCGATCTCATCCTTGATTTGCTATTTAAAC.

Constructs were injected into *M{Act5C‐Cas9.P.RFP‐}ZH‐2A DNAlig4^169^* flies (Bloomington stock #58492) and selected for dsRed‐positive flies. Positive candidates were confirmed by sequencing.

### 4.9 qRT‐PCR analysis of *shot* ^Δ^ *^PRR^* mutant embryos

For RNA isolation, at least 10 *Drosophila* third instar larvae were placed in Trizol (Invitrogen) and homogenized using a pestle. Total RNA was isolated using the NucleoSpin RNA II kit (Macherey & Nagel), and RNA concentration was analyzed via a NanoDrop spectrophotometer (Thermo Scientific). For first strand cDNA synthesis, 500 ng of total RNA was transcribed using the QuantiTect RT Kit (Qiagen). Real‐time PCR was performed with 1 μl cDNA per reaction using the Power SYBR Green PCR Master Mix (ThermoFischer Scientific) as detection dye. Experiments were performed with the BioRad C1000 Thermal Cycler. cDNA samples were run in triplicate, and the average CT was used to analyze the expression levels via the −2ΔΔCT method. Experiments were repeated with independently isolated RNA samples. Actin 5C (Act5C, act) and Ribosomal protein L32 (RpL32, rp49) were used as reference genes. Expression analysis was performed using BioRad C1000 System software and GraphpadPrism. The following oligonucleotides were used for real time PCR analysis (Figure S7a):

- Ctail (recognizes almost all isoforms): fw – GGTCCCATCATCAAGGTACG; rev – CATGGCTACCCTCGTTGTC.
- SRR (recognizes all isoforms): fw – ACTGAAGGAACAATGGACTCG; rev – CCAGAAAGAAGCAAAGCCTC.
- PRR1 (recognizes only PRR): fw – TCTACACCACTACCTACAGCA; rev – CAAGCCATCGCTACTATAGACG.
- CH2 (recognizes all isoforms): fw – GAAGTATCCCGTCCACGAG; rev – ACCACTCAATGTGCTCCTG.
- CH2 (recognizes only A*‐ and B*‐type isoforms; Figure 1a): fw – CACCATCATCAGAGCTACCA; rev – CGTTCCATTGTTGCCACC.

### 4.10 Sequencing the *shot^V104^* breakpoint

The chromosomal breakpoint of *shot^V104^* was described to be in a 373 bp region between 73,398 and 73,771 bp of the *shot* locus (Strumpf & Volk, 1998). We used an inverse PCR approach to determine the exact chromosomal break‐point of *shot^V104^*. For this, genomic DNA of 200 homozygous *shot^V104^* embryos was isolated (Berkeley *Drosophila* Genome Project protocol; https://www.fruitfly.org/about/methods/inverse.pcr.html) and restricted with Sau96I. The restricted DNA was purified, diluted 10:1, and ligated into circular fragments. Using primer pairs designed to face toward the unknown region covering the breakpoint (fw: CCTGCTTTCAAACTAACATCCTGC; rev: CTGGCTGAATGGCAATTAAAGG), the circular DNA fragment containing the *shot^V10^* ^4^ breakpoint region was amplified using a High Fidelity PCR Kit (Eppendorf and Roche). PCR products were gel‐extracted, cloned into pDrive (Qiagen), and sequenced. The sequencing of one inverse PCR fragment showed a perfect alignment with wild‐type genomic DNA until bp73.681 followed by an adenine and thymine‐rich region. Using BLAST (flybase.org), we identified this region as part of the centromeric region of chromosome 2R (Figure 7). The breakpoint was confirmed via PCR (Figure 7) using the following sequence‐specific primers:

- forward sense primer: TCTACGCTTGCGCTGCCCGCTCGCC (binding the wild‐type *shot* region 100 bp upstream of the breakpoint);
- reverse antisense wt1: TTTGTACGCATTGGCATGGCAGATG (binding the wild‐type region before the breakpoint);
- reverse antisense wt2: GGCAGATGCACAGATGCATTTATATACGC (binding the wild‐type region directly after the break point);
- reverse antisense mutant 1: TGTTAGTTCTTATACAAGAAGATTCAATAAATAAAAGC (in the putative new *shot^V104^* sequence after the breakpoint).

## CONFLICT OF INTEREST

The authors declare no conflict of interest.

## AUTHOR CONTRIBUTIONS

Cell culture experiments, image analysis, and cloning of constructs: Yue Qu and Kriti Gupta. Embryo analyses: Juliana Alves‐Silva. Breakpoint analysis and CRISPR design: Ines Hahn. Cloning of constructs, qRT‐PCR, and developing transfection procedure: Jill Parkin. Cell culture experiments and image analysis: Natalia Sánchez‐Soriano. Electron microscopy, embryo analyses, image analysis, and writing the manuscript including figure compositions: Andreas Prokop.

## Supporting information

Figure S1. Impact of Cyto D on Shot‐PE localisation. Primary neurons at 6–8 HIV on glass, treated with DMSO (control, **A**) or Cyto D (**B**) and stained for GFP (green), tubulin (red) and actin (blue); grayscale images on the right show single channels as indicated; asterisks indicate cell bodies, arrowheads the tip of axons; scale bar in A represents 10 μm in all images. For similar results compare Fig.6E,F.

Figure S2. Comparison of the CH domains of Shot and human α1‐actinin. Sequences are taken from the ACTN1‐203 isoform (ensembl.org: ENST00000394419.9) and the Shot‐PE isoform (flybase.org: FBtr0087618) and were aligned using UniProt Align (www.uniprot.org/align); asterisks indicate identical residues, dots and colons similar residues. **A**) Alignment of the first (CH1, black) and second (CH2, blue) CH domain of Shot with the first CH domain of hACTN1 indicates a high similarity of CH1 but considerable deviation of CH2 from the prototype CH domain; structurally important residues are colour‐coded and the actin‐binding consensus is provided above in green, as detailed elsewhere (Yin et al., 2020). **B**) Alignment of both CH domains as they occur in tandem in Shot and hACTN1 indicates a higher degree of identity and similarity of the two second CH domains (blue) to one another, than to the first CH domain (as shown for Shot CH2 in A).

Figure S3. Only the Life‐containing N‐terminus shows strong F‐actin association. **A‐D**) Primary neurons at 24 HIV on ConA transfected with GFP (controls) or N‐terminal constructs as indicated on the left (colour code as used in Fig.1); grayscale images on the right show single channels, as indicated; asterisks indicate cell bodies, arrowheads the tip of axons; scale bar in A represents 15 μm in all images. **E**) Bars correspond to experiments shown on the respective left and indicate the frequency of neurons with GCs that contain spools; number of neurons analysed are shown in orange, the percentage of neurons with GCs that contain spools in black in the bar, and P‐values obtained via Chi^2^ tests on top of bars. Data were normalised to wild‐type controls performed in parallel to all experiments (dashed red line).

Figure S4. Animated GIF showing further examples of phenotypes induced by Shot‐PE‐Life::GFP expression. Primary neurons at 6–8 HIV on glass with *scabrous‐Gal4*‐induced expression of Shot‐PE‐Life::GFP, stained for tubulin (green), actin (red) and GFP (blue); the animation sequence shows single channels as grayscale images, as indicated top left in animation steps. Symbols indicate the following: asterisks, cell bodies; arrowheads, MT bundle split; arrows, ‘tennis racket’ spools; white curved arrows, unusual MT bundle malformations; open curved arrows, unusually bundled MTs in cell bodies. View or download: https://figshare.com/articles/figure/FigS4‐Qu_al_gif/17056364.

Figure S5. Filopodia data accompanying analyses shown in Fig.4B‐E. **A)** Schematics on the left summarise the key findings for wild‐type, *shot^3/3^*, *chic^221/221^* or *shot^3/3^ chic^221/221^* mutant primary neurons at 6–8 HIV on glass; indicated are: axon length (relative to black dashed line), filopodia number (black dots), filopodial length (white/open arrows) and MT disorganisation (curved arrows). **B,C**) Quantifications of filopodial lengths (B) and numbers of filopodia per neuron (C); numbers of analysed filopodia (B) or neurons (C) are indicated in orange; median values in blue, and P‐values obtained via Mann–Whitney rank sum tests in black/grey; data were normalised to wild‐type controls performed in parallel to all experiments (dashed red lines). Note that reduced filopodia numbers are due to regulatory roles of Shot in actin nucleation (Sánchez‐Soriano et al., 2009), and shorter filopodia due to promoting roles of profilin/Chic in actin polymerisation (Gonçalves‐Pimentel et al., 2011); both mechanisms are independent and therefore fully penetrant in the double‐mutant constellation.

Figure S6. Phenotypes of *shot^V104^* mutant embryos. All images are taken from late stage 17 embryos of wild‐type controls (wt; left), strong *shot* mutant alleles (HG25, sf20/3, sf20, middle; Lee et al., 2000; Prokop et al., 1998), and *shot^V104^* mutant embryos (right). **1^st^ row**: Fascilin 2‐stained (Fas2) ventral nerve cords (part of the CNS; white arrow pointing at the most lateral longitudinal fascicle); only strong Shot deficiency causes the upregulation of Fas2 in nerve roots (open white arrow; Bottenberg et al., 2009; Prokop et al., 1998). **2^nd^ row**: flat dissected embryos stained for the synaptic marker Synaptotagmin (Syt; white arrowheads pointing at neuromuscular synapses); stained dots are severely reduced only by strong Shot deficiency (open arrowhead; Löhr et al., 2002). **3^rd^ row**: a detail of the ventral nerve cord expressing the membrane marker CD8::GFP driven by *eve^RN2^‐Gal4* in a subset of motor neurons; somata (S), dendrites (D) and axons (A) are indicated: dendrites reduced only upon strong Shot deficiency (Bottenberg et al., 2009; Prokop et al., 1998). **4^th^ row**: flat dissected embryos expressing actin::GFP driven by *stripe‐Gal4* (*sr*) in epidermal tendon cells (anchoring cells where muscles attach); only in the wild‐type do tendon cells have their usual cell shapes (white curved arrow), whereas tendon cells become stretched (open curved arrows) upon strong Shot deficiency and in *shot^V104^*, indicating defects of the muscle‐tendon junction (MTJ; Alves‐Silva et al., 2008). **5^th^ & 6^th^ row**: micrographs of MTJs, central parts of which are shown twofold enlarged below; electron‐dense MTJs (indicated by double chevrons) between muscles (m) and tendon cells (t) are properly formed in all genotypes, but the sub‐membranous electron dense layer on the tendon cell side (black arrow in wt) is much thinner in the two *shot* mutant conditions (open arrows), and the characteristic MT arrays (arrowhead in wt) are absent, indicating the *shot*‐specific tendon cell rupture phenotype (Prokop et al., 1998). This MTJ rupture in *shot^V104^* embryos is consistent with reports that Shot‐PE‐∆Ctail cannot rescue *shot* mutant tendon cell phenotypes (Alves‐Silva et al., 2012), and that the Shot‐PH isoform is not enriched in this cell type (Röper and Brown, 2003). Scale bar in A represents 20 μm in 1^st^, 40 μm in 2^nd^, 7 μm in 3^rd^, 30 μm in 4^th^, 1.2 μm in 5^th^ and 0.6 μm in 6^th^ row.

Figure S7. Generation and analysis of *shot^∆PRR^*. **A**) Normalised data from quantitative RT‐PCR analyses of embryos from wild‐type and two independent CRISPR/Cas9 mutant lines (*shot^∆PRRa^*, *shot^∆PRRb^*) using probes against different exons (red lines) encoding different functional domains (indicated below graphs); they suggest that the PRR is deleted in the mutant strains, but that both versions of the allele cause severe expression changes of other exons suggestive of splice aberrations. **B**) A list of different splice variants of *shot* (modified from Voelzmann et al., 2017): names provided on the left and right, colour‐coded as in Fig.1A and sorted by their A*‐,B*‐,C*‐ and D*‐type N‐termini (compare Fig.1A). **C**) Schematic representation of the *shot‐RH* genomic sequence from exon 11 to 14 (position in the *shot* gene indicated by red lines) aligned with sequences from other *Drosophila* species (https://genome.lbl.gov/vista/customAlignment.shtml; introns in pink, exons in blue); the amplitude indicates the degree of evolutionary conservation, thus identifying areas that are not well‐conserved and therefore suitable deletion sites less likely to affect important splice sites. Black arrows indicate the locations of the two guide RNAs for CRISPR/Cas9 incision (slightly removed from the 5' end, and at the very 3' end of exon 12), black bars the location of PCR‐amplified flanking regions (covering part of intron 10/11 up to the end of intron 11/12; start of intron 12/13 to the start of exon 14) cloned into 5' and 3' multiple cloning sites (MCS) of *pHD‐DsRed‐attP* (D; vector scheme adapted from)(Gratz et al., 2014) using EcoRI/NotI and BglII/PstI restriction sites (for details see methods).

## Data Availability

The data used for our analyses will be made available on request from the authors.
