# Supplementary figures and images for: Re‐evaluating the actin‐dependence of spectraplakin functions during axon growth and maintenance
Source: Dev Neurobiol. 2022 Apr 22;82(4):288–307. doi: 10.1002/dneu.22873 (PMC9320987; doi:10.1002/dneu.22873)

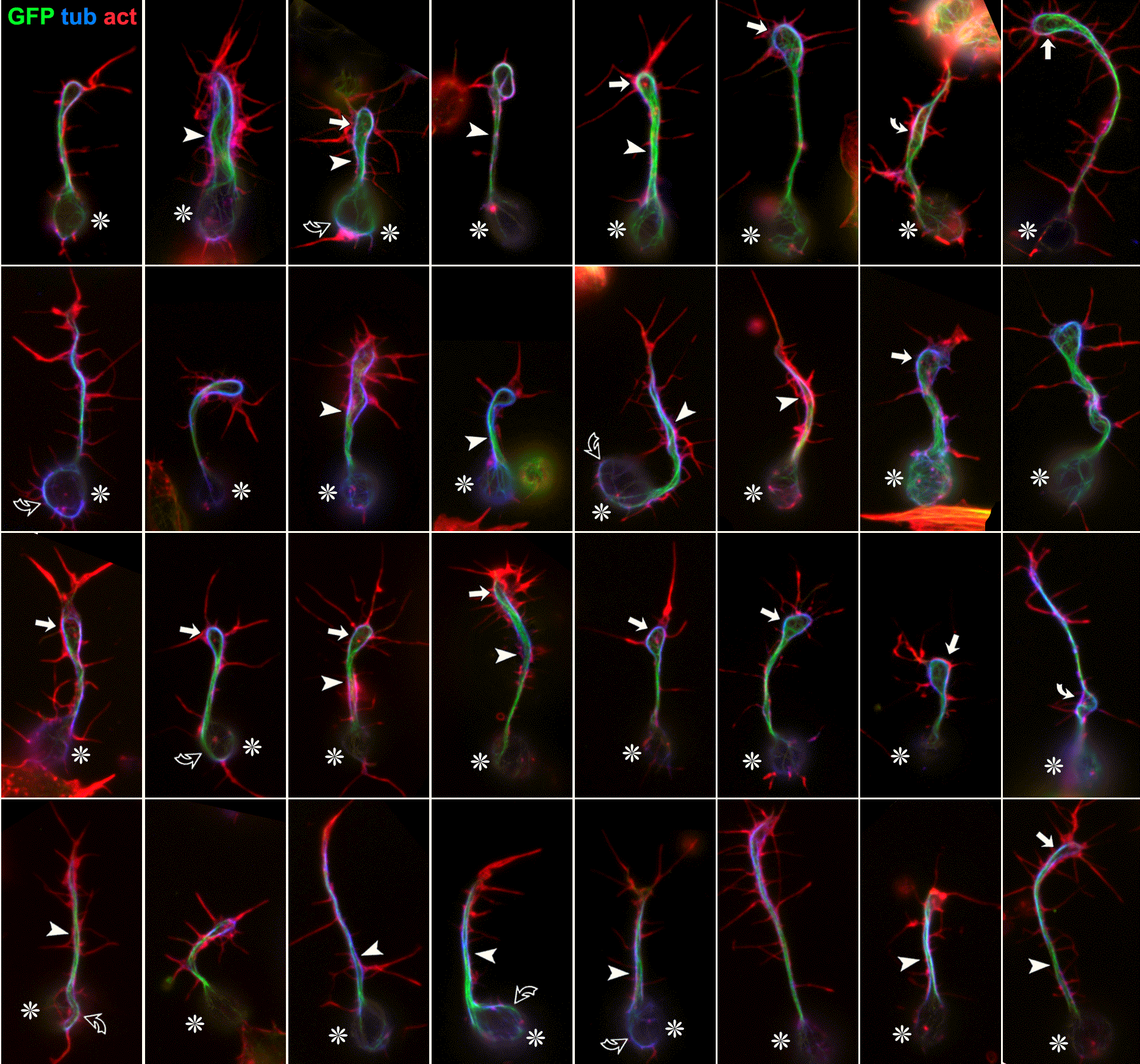

Supplement: Supplementary file 4 — Figure S4. Animated GIF showing further examples of phenotypes induced by Shot‐PE‐Life::GFP expression. Primary neurons at 6–8 HIV on glass with scabrous‐Gal4‐induced expression of Shot‐PE‐Life::GFP, stained for tubulin (green), actin (red) and GFP (blue); the animation sequence shows single channels as grayscale images, as indicated top left in animation steps. Symbols indicate the following: asterisks, cell bodies; arrowheads, MT bundle split; arrows, ‘tennis racket’ spools; white curved arrows, unusual MT bundle malformations; open curved arrows, unusually bundled MTs in cell bodies. View or download: https://figshare.com/articles/figure/FigS4‐Qu_al_gif/17056364. [file DNEU-82-288-s006.gif]
